# Decoupled level and flow rate control of a two-tank system in beverage production: A comparative analysis of Fuzzy-PID and GA-PID for minimum time operation

**DOI:** 10.1371/journal.pone.0317600

**Published:** 2025-02-05

**Authors:** Tamiru Demelash Kassie, Mebratu Sintie Geremew, Kassahun Ashagrie Chanie

**Affiliations:** Department of Electrical & Computer Engineering, College of Engineering and Technology, Mizan-Tepi University, Tepi, Ethiopia; Ningbo University, CHINA

## Abstract

Due to the nonlinear characteristics of the valves and the interactions between the controlled variables, designing a control system for coupled tanks is a difficult task. This paper deals with the comparative study between Fuzzy-PID and GA-PID controllers for decoupling level and flow rate control of two tank systems for beverage factories with minimum time optimal operation. In most process control industries, each process requires multiple control variables. Here two input two output (TITO) systems are considered highly interacting multivariable control systems. The decoupling control scheme (Pre-compensator (dynamic) decoupling) is used to reduce the correlation between the controlled and manipulated variables by diagonalizing the system. The two independent SISO systems are further controlled by different controllers so that the system can trace the set point and yield a good time response. Two radically different control approaches are presented and compared for this system’s dynamics, motivated by a desire to provide precise liquid-level control and regulate the flow rate. MATLAB /Simulink model and tuning algorithm (GA) are used for simulation. As the simulation result ensured, the GA-PID controller is the used for the specified system which is based on the transient and steady-state specifications. Quantitatively; the GA-PID controller has 39.167ms rise time, 8.50sec settling time, and -0.393% overshoot; whereas FLC-PID has 118.101ms rise time, 8.65sec settling time, and -0.033% overshoot. But in GA with PID controllers, the external disturbance tolerance capability of the proposed scheme, meaning robustness against external disturbance has a slight difference and FLC-PID has perfectly achieved the robustness. Depending on the result, FLC-PID has more result than GA-PID controller based on the set of specifications. Hence robustness is more important than time performances.

## 1. Introduction

This paper is concerned with modeling, analysis, and control of coupled tank systems. The goal is to develop advanced control strategies capable of handling the complex dynamics of the coupled tank system for beverage factories. Applying the decoupling technique while seeking to control the liquid level and to regulate the flow rate at the desired value with minimum time.

### 1.1 Background

Water purification, chemical, and biochemical processing, automatic liquid dispensing, food and beverage processing, and pharmaceutical industries all use liquid level control as a common example of process control. Control quality has a direct impact on product quality and equipment safety. The coupled tank liquid level control system, on the other hand, has a long lag, is nonlinear, and is complex, and the control accuracy is directly influenced by system status, system parameters, and the control algorithm. In the process industry, level control is one of the most important control system variables [1].

Coupled tank level control is difficult for a variety of reasons. Because of the valves, it is a highly nonlinear problem. Second, the interactions between process variables can exacerbate the control problem. Furthermore, according to Conventional feedback controllers may not be able to control the system properly because the interconnected tanks have a delay in response [2].

The majority of industrial process control applications have multiple input and output variables, which is referred to as a MIMO system. Heat exchangers, chemical reactors, and distillation columns are all examples of MIMO systems. Performing operations on a MIMO system is more difficult than on a SISO system. The interaction between the input and output variables is the reason for this [3].

A coupled tank system for liquid level control is a type of plant that is commonly found in commercial ventures, particularly in the chemical process industry. Nonlinearity exists in the coupled tank system. The reason for the non-linearity is that the transfer function changes as the height of the liquid in a tank changes, and the controller should be able to adapt to these changes. System dynamics and interacting nature are the issues with level control in coupled tank processes. As long as the inflow rate, i.e., the inlet and outlet flow rates, remain constant, the level will remain constant [4].

**Loading** is a common term used to describe interaction. If each input affects more than one output or if a change in one output affects the other outputs, the process is said to be interacting; otherwise, it is said to be non-interacting [5].

We must apply a new element called a **Decoupler** into the control system while designing two strongly interacting loops. Decouplers are used to cancel the interaction effects between two loops, resulting in two non-interacting control loops. To put it another way, the main goal of a decoupling control system is to eliminate complex loop interactions so that a change in one process variable does not result in corresponding changes in the other process variables [6].

The inlet flow rate, which is the variable used to bring the process variable to its set point, is the manipulated variable. Food processing, water purification systems, filtration, pharmaceutical industries, decoration, boilers, dairy, beverage, nuclear power generation plants, industrial chemical processing, spray coating, and other industrial applications all require liquid-level control. Typical actuators, such as electrical pumps and motorized valves, are widely used for feedback control, and many types of level sensors, such as displacement floats, pressure sensors, and so on, provide liquid level measurement [7].

This paper has made main contributions:

- ➢ To model coupled tank system dynamics using state space modeling technique and validate the model.
- ➢ To design a decoupling controller that can successfully decouple a TITO-coupled tank system.
- ➢ To design a fuzzy-PID controller/ to tune the parameters of the PID controller with FLC and implement it in the TITO system.
- ➢ To design a GA-PID controller.
- ➢ To control the desired level of tanks and regulate the flow rate with minimum time optimal operation and to compare the performance of the controllers.

## 2. Mathematical modeling of coupled tank system

### 2.1 Introduction

**Fig 1:**
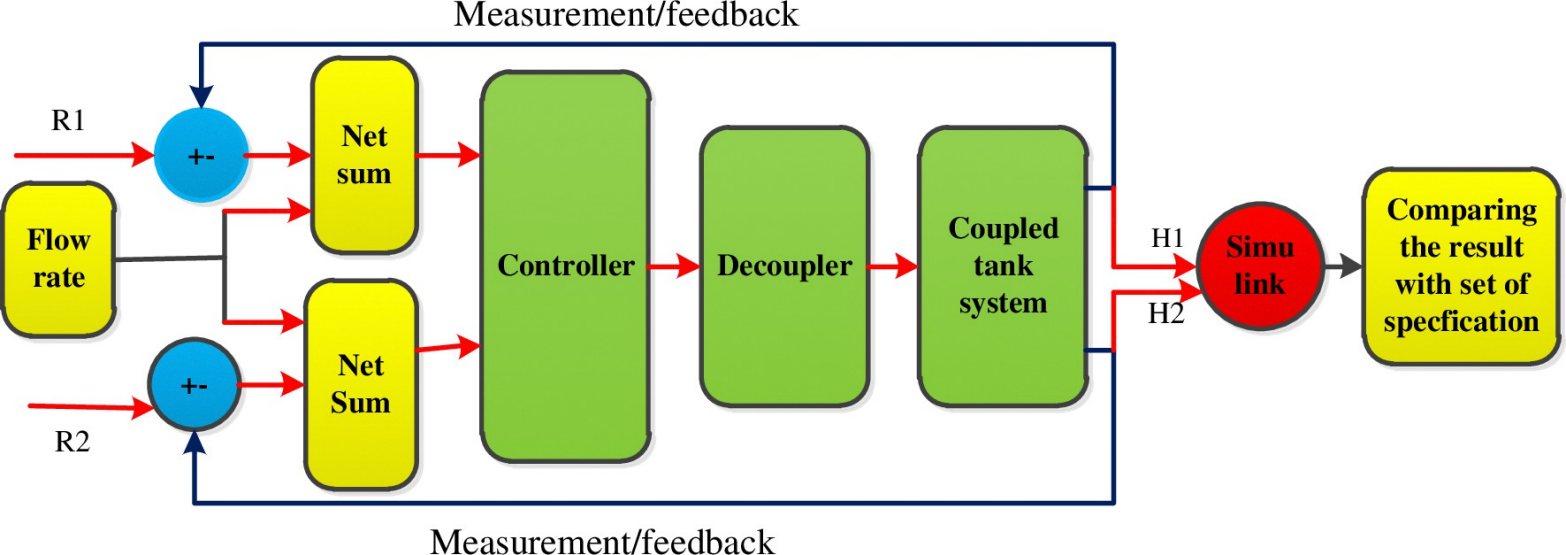
Block diagram of the overall system.

The methodology involves two port block diagrams with two blocks: the plant and the controller. The diagram in Fig 1 includes reference inputs (R1 and R2), manipulated variables, controllers, tank systems, decoupler, and actual outputs. Simulink is used to analyze system performance and identify the controller.

**Fig 2:**
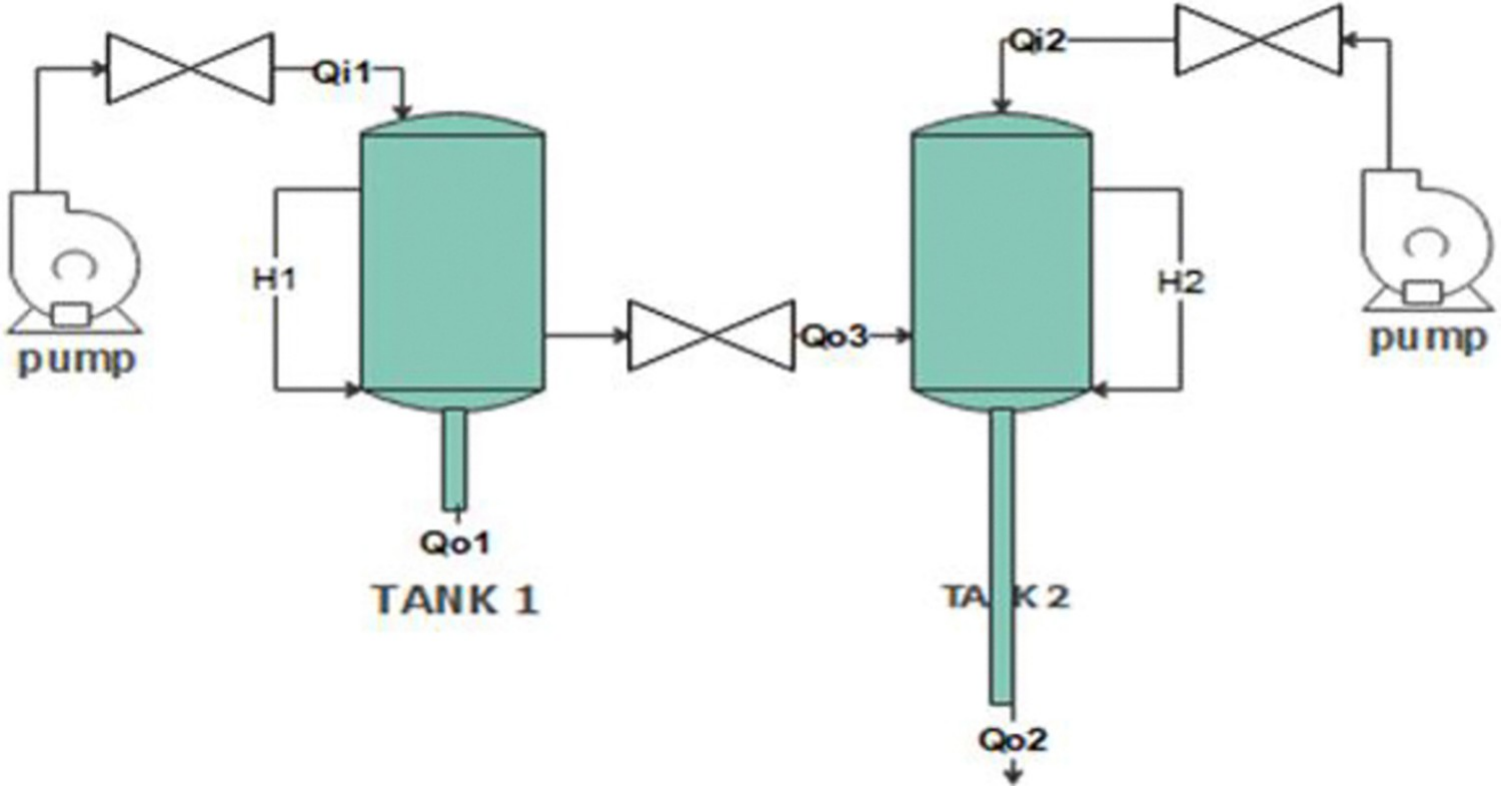
Schematic diagram of coupled tank system.

It is critical to understand the mathematics of how the coupled tank system behaves before beginning the controller design process. Nonlinearity in the dynamic model has been observed in this system. As shown in **Fig 2** the control goal is to manipulate Q_i1_ and Q_i2_ liquid flows to regulate the liquid levels of H_1_ and H_2_ in the two tanks.

The rate of change of fluid volume in each tank equals the net flow of fluid into the tank, according to a simple mass balance. As a result, the dynamic equation for Tank 1 and Tank 2 is as follows:

- ➢ Rate of change of volume in tanks = flow in–flow out

$$\frac{dV}{dt}=Qin-Qout$$ (1)

Since volume is (area x height):

$$A1(\frac{dH1}{dt})=Qi1-Q01-Q03\;\&\;A2(\frac{dH2}{dt})=Qi2-Q02+Q03$$ (2)

Where; H_1_, and H_2_ are the heights of the fluid in tanks 1 and 2, respectively, A_1_ = cross-sectional area of tank 1 and A_2_ = cross-sectional area of tank 2, Qo_3_ = fluid flow rate between tanks Pump flow rates into tanks 1 and 2 are represented by Q_i1_ and Q_i2_, respectively, Qo_1_ and Qo_2_ are the fluid flow rates out of tank 1 and tank 2, respectively.

A simple orifice can be used to model each outlet drain. The outlet flows in each tank are proportional to the square root of the head of liquid in the tank, according to Bernoulli’s equation for steady, non-viscous, incompressible fluids. The flow between the two tanks is proportional to the square root of the head differential in the same way.


$$Q01=\beta 1\sqrt{H1};Q02=\beta 2\sqrt{H2};Q03=\beta 3\sqrt{\left(H1-H2\right)}$$ (3)

Where *β*1,*β*2, *and β*3 are proportional constants that depend on the coefficients of discharge, the cross-sectional area of each orifice, and the gravitational constant.

Substituting Eq (3) into Eq (2), a set of nonlinear state equations that describe the system dynamics of the coupled tank are derived.


$$A1(\frac{dH1}{dt})=Qi1-\beta 1\sqrt{H1}-\beta 3\sqrt{\left(H1-H2\right)}$$ (4)

$$A2(\frac{dH2}{dt})=Qi2-\beta 2\sqrt{H2}+\beta 3\sqrt{\left(H1-H2\right)}$$ (5)

For a set of inflows *Qi*1 and *Qi*2 the fluid level in the tanks is at some steady state level H_1_ and H_2_. Consider a small variation in each inflow, q_1_ in Q_i1_ and q_2_ in Q_i2_. Let the resulting perturbation in level be h1 and h2 respectively. From Eqs (4) and (5), the equation becomes:

For Tank 1:

$$A1(\frac{d(H1+h1)}{dt})=(Qi1+q1)-\beta 1\sqrt{\left(H1+h1\right)}-\beta 3\sqrt{\left(H1-H2)+(h1-h2\right)}$$ (6)

For Tank 2:

$$A2(d\frac{\left(H2+h2\right)}{dt})=(Qi2+q2)-\beta 2\sqrt{\left(H2+h2\right)}+\beta 3\sqrt{\left(H1-H2)+(h1-h2\right)}$$ (7)

Subtracting Eqs (4) and (5) from Eqs (6) and (7), the equations obtained are as follows:

$$A1(\frac{dh1}{dt})=q1-\beta 1(\sqrt{\left(H1+h1\right)}-\sqrt{H1})-\beta 3(\sqrt{\left(H1-H2+h1-h2\right)}\sqrt{\left(H1-H2\right)})$$ (8)

$$A2(\frac{dh2}{dt})=q2-\beta 2(\sqrt{\left(H2+h2\right)}-\sqrt{H2})+\beta 3(\sqrt{\left(H1-H2+h1-h2\right)}\sqrt{\left(H1-H2\right)})$$ (9)

For small perturbations:

$$\sqrt{\left(H1+h1\right)}=\sqrt{\left(H1(1+\frac{H1}{2H1})\right)}\;\&\;\sqrt{\left(H2+h2\right)}-\sqrt{H2}\approx \frac{h2}{2\sqrt{H2}}$$ (10)

Therefore;

$$\sqrt{\left(H1+h1\right)}-\sqrt{H1}\approx \frac{h1}{2\sqrt{H1}}\;\&\;\sqrt{\left(H2-H1+h2-h1\right)}-\sqrt{\left(H2-H1\right)}\approx \frac{h2-h1}{2\sqrt{\left(H2-H1\right)}}$$ (11)

In Eqs (10) and (11), note that the coefficients of the perturbations in level are functions of the steady state operating points H_1_ and H_2_. Note that the two equations can also be written in the form:

$$\left(\frac{dh1}{dt})=\frac{1}{A1}(q1-q01-(\frac{\beta 3\sqrt{\left(H1-H2\right)}}{2})(h1-h2)\right)$$ (12)

$$\left(\frac{dh2}{dt})=\frac{1}{A2}(q2-q02+(\frac{\beta 3\sqrt{\left(H1-H2\right)}}{2})(h1-h2)\right)$$ (13)

Where; q_o1_ and q_o2_ represent perturbations in the outflow at the drain pipes. This is appropriate in the case where the outflow is controlled by attaching an external clamp for instance. Each value of β_1_, β_2_, β_3_, A_1_, A_2_, H_1,_ and H_2_ can be obtained from mathematical modeling equations as shown in Table 1.

**Table 1:** Parameter values [8].

H_1_, H_2_	30 m, and 20 m respectively
*β*1	50
*β*2	50
*β*3	50
*A*1	400 m^2^
*A*2	400 m^2^

By using the Parameters value and Eqs (13) and (14), we can get the following equations in the form of manipulating variables q_1_, q_2_ and process variables h_1_, h_2_:

$$\frac{dh1}{dt}=0.0025(q1-4.56h1-79(h1-h2))$$ (14)

$$\frac{dh2}{dt}=0.0025(q2-5.59h2+79(h1-h2))$$ (15)

Rearranging into state space representation as follows:

$$\left[\begin{array}{c} \overset{•}{h1}\\ \overset{•}{h2} \end{array}]=[\begin{array}{cc} -0.184 & 0.198\\ 0.198 & -0.212 \end{array}][\begin{array}{c} h1\\ h2 \end{array}]+[\begin{array}{cc} 0.0025 & 0\\ 0 & 0.0025 \end{array}][\begin{array}{c} q1\\ q2 \end{array}\right]$$ (16)

$$\left[\begin{array}{c} y1\\ y2 \end{array}]=[\begin{array}{cc} 1 & 0\\ 0 & 1 \end{array}][\begin{array}{c} h1\\ h2 \end{array}]+[\begin{array}{cc} 0 & 0\\ 0 & 0 \end{array}][\begin{array}{c} q1\\ q2 \end{array}\right]$$ (17)

Above Eqs (16) and (17) are the transfer function of coupled tank system in the form of state space matrices, where:

$\overset{•}{h1}$ = Derivative of state variable for tank 1

$\overset{•}{h2}$ = Derivative of state variable for tank 2

*h*1 = State variable for tank 1

*h*2 = State variable for tank 2

*q*1 = Input variable for tank 1

*q*2 = Input variable for tank 2

*y*1 = Output variable for tank 1

*y*2 = Output variable for tank 2

From the above analysis, I have found state space matrix A, B, and C &Results in the following matrix transfer function:

G(S)=[0.0025*S+5.3*e^−4S^2+0.396*S−0.0001964.950.018*S^2+0.0072*S−0.000664.950.018*S^2+0.0072*S−0.000660.0025*S+4.6*e^−4S^2+0.396*S−0.000196]

### 2.2 Controllers theory and general facts

#### 2.2.1 PID (Proportional-Integral-Derivative) controller

PID controllers are a popular choice in the process industry for their simplicity, rapid response, and ability to eliminate steady-state errors [9]. Their low cost and effectiveness in handling complex, nonlinear systems make them a versatile tool for industrial applications.

**Fig 3:**
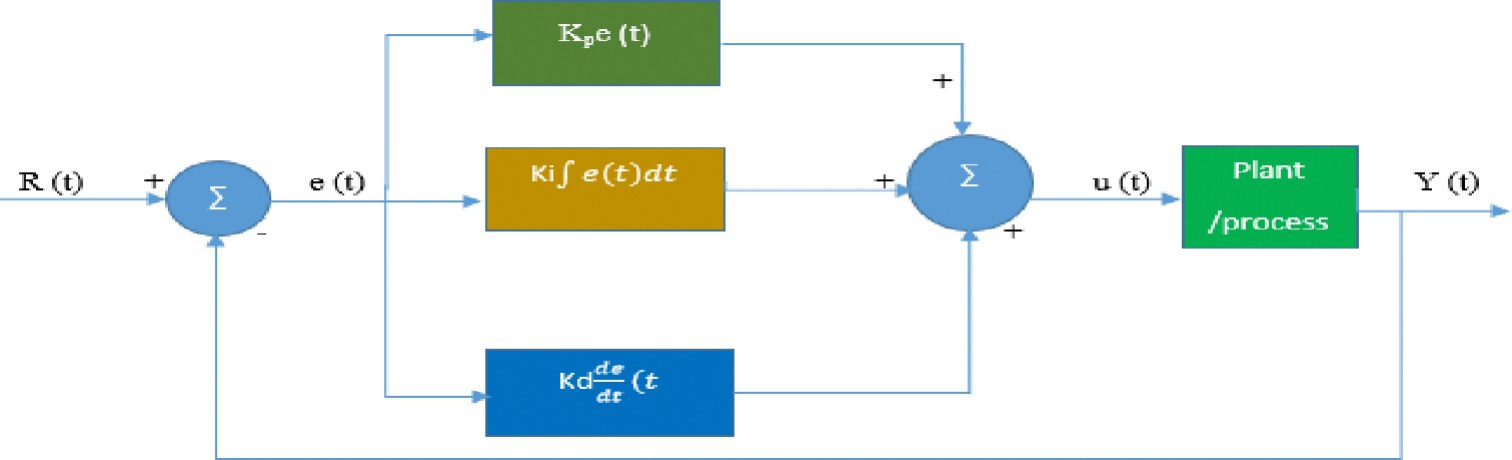
PID (Proportional Integral Derivative) controller structure.

PID controllers work by calculating and applying a corrective output to adjust the process, ensuring that the actual output matches the desired setpoint. **The overall controller structure of PID is shown in Fig 3.** This feedback mechanism helps maintain accurate and stable process control.


$$u(t)=Kpe(t)+Ki\int e(t)dt+Kd\frac{de(t)}{dt}$$ (18)

$$u(t)=Kp[e(t)+\frac{1}{Ti}\int e(t)dt+Td\frac{de(t)}{dt}]$$ (19)

$$\mathrm{Where};\;Ki=\frac{Kp}{Ti};Kd=KP•Td$$ (20)

The proportional term **P** provides a control action based on the error signal, the integral term **I** produces steady-state errors, and the term "**D**-improving transient response.

**PID-Controller-**PI-controller cannot predict future error behavior, so it reacts normally. The D-controller anticipates error behavior, increasing system response and stability. Combining these controllers improves response speed and compensates for phase lag caused by the I-controller. The individual effects of these three terms on the closed-loop performance are summarized in Table 2

**Table 2:** Effects of P, I, and D individual controllers.

Closed-loop system	Rise time	Overshoot	Settling time	Steady-state error	Stability
Increasing Kp	Decrease	Increase	Small increase	Decrease	Degrade
Increasing Ki	Small decrease	Increase	Increase	Large decrease	Degrade
Increasing Kd	Small decrease	Decrease	Decrease	Minor change	Improve

#### 2.2.2 Fuzzy Logic Controller (FLC)

Fuzzy logic controllers (FLCs) use fuzzy set theory to represent human operator experience in linguistic variables called fuzzy rules. Although this article was first published in 1965, the use of fuzzy logic (FL) increased after the second half of the 1970s when two more articles, in which the application of fuzzy set theory to uncertain systems and decision-making were described. FL applications have been gaining a high speed ever since the Japanese started using them in commercially available appliances. Nowadays, it is possible to find fuzzy-based applications in almost every area [10].

Fuzzy logic controllers (FLCs) based on fuzzy set theory are used to represent the experience and knowledge of a human operator in terms of linguistic variables called fuzzy rules. Since an experienced human operator adjusts the system inputs to get a desired output by just looking at the system output without any knowledge of the system’s dynamics and interior parameter variations, the implementation of linguistic fuzzy rules based on the procedures done by human operators does not also require a mathematical model of the system.

#### 2.2.3 Fuzzy logic controller structures

**Fig 4:**
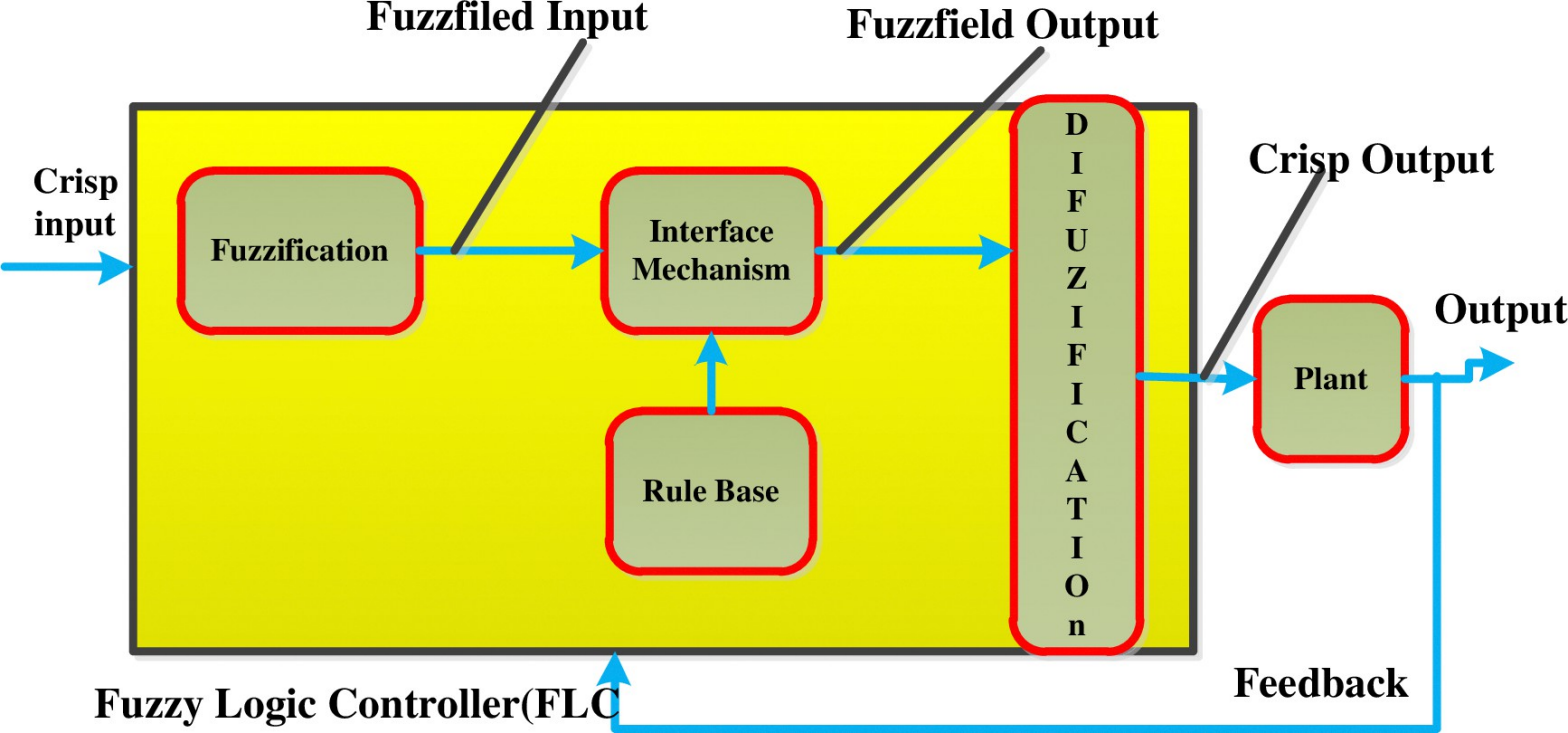
Fuzzy logic controller general structure.

Fig 4 below shows the general structure of the fuzzy interface system. Fuzzy logic control can play an important role because knowledge-based design rules can easily be implemented in systems with unknown structures.

### 2.3. Genetic algorithms

Genetic Algorithm (GA) is an evolutionary computing technique that uses Darwin’s Theory of Evolution’s concept of ’Survival of the Fittest’ to find the global optimum solution for optimization problems. GA assumes unique solutions are described by parameters, with the fitness function reflecting the candidate’s goodness. The fittest chromosome produces high-quality offspring, resulting in a more solution [11].

**Fig 5:**
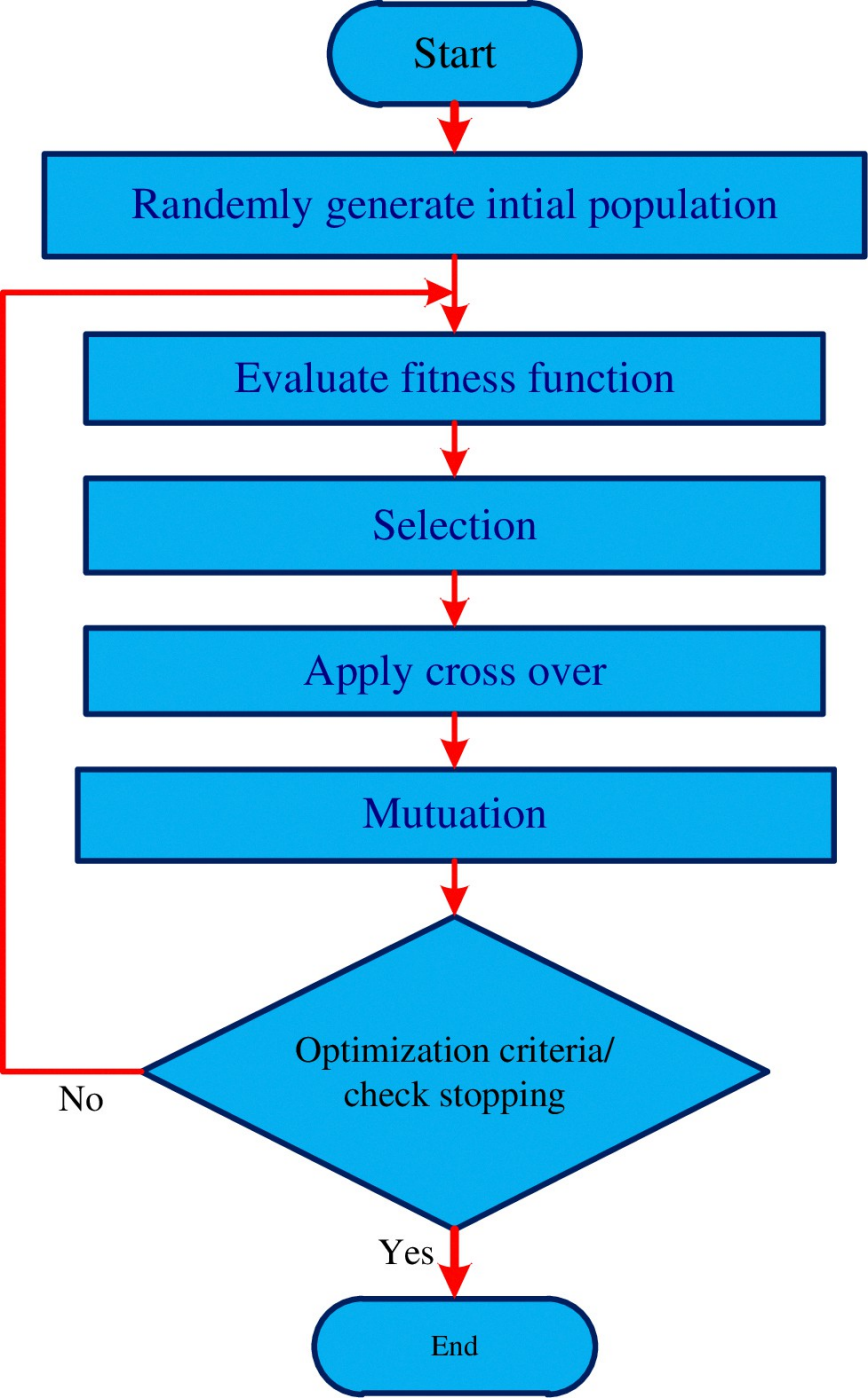
Block diagram of GA/ flow chart of genetic algorithm.

Initialization of the GA parameters and flow chart is as shown in Fig 5:

## 3. System design and simulation result

### 3.1. Introduction

This part discusses controller designs, performance evaluation parameters, Simulink model simulations, and performance comparisons between conventional PID with FLC and GA with PID, using figures and performance specifications.

### 3.2. Design decoupler

**Fig 6:**
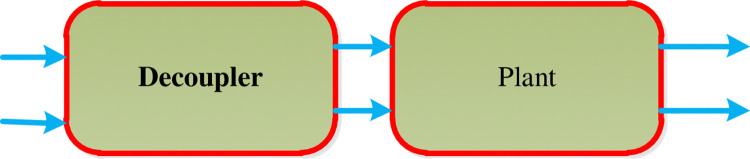
Decoupler block diagram.

A decoupling control scheme minimizes interaction between controlled and manipulated variables by diagonalizing the system and decomposing input and output processes by two decentralized SISO controllers. This MIMO control is achieved through state measurement via a feedback law, allowing for better time response and traceability. the overall block diagram of the decoupler is shown in Fig 6.

Pre-compensators or pre-post compensators can be used to decouple multivariable systems, typically at a given frequency, ensuring good system performance at the frequency of decoupling.

#### 3.2.1 Decoupling design technics (pre-compensator method)

**Fig 7:**
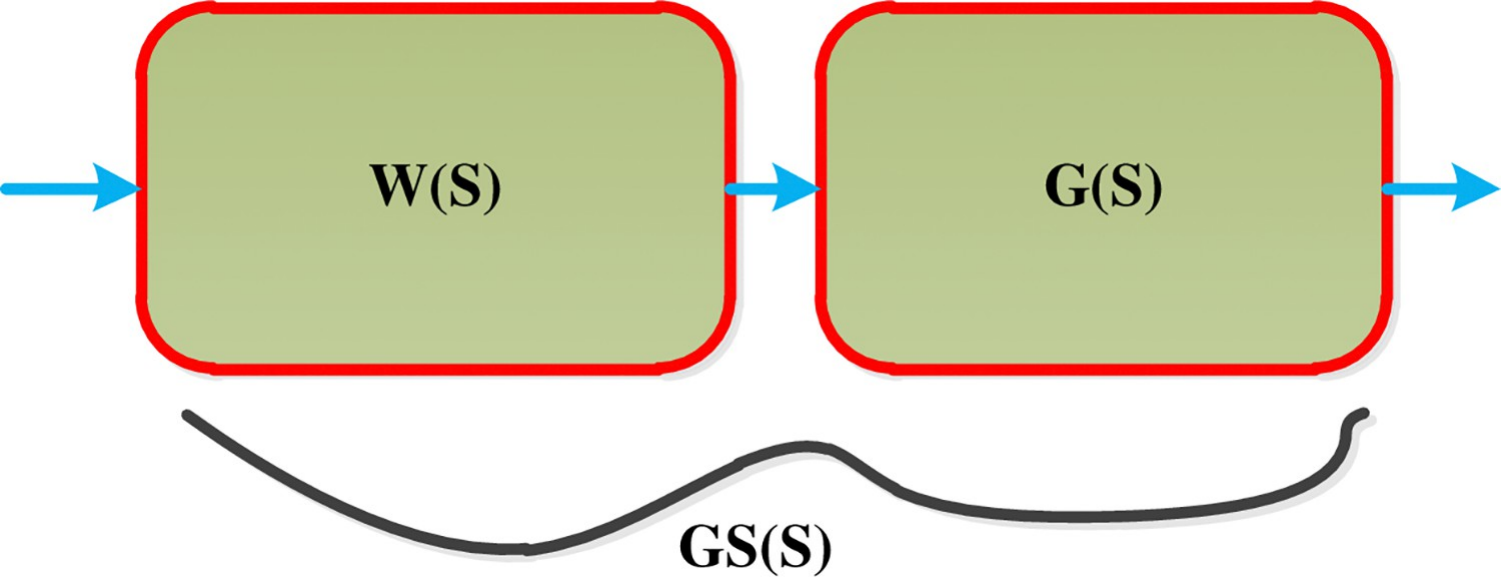
Pre-compensator arrangement.

The pre-compensation approach employs three methods: dynamic decoupling, steady-state decoupling, and approximate decoupling, with the primary goal of decoupling the system. The resulting system W(s) G(s) is shaped “approximately-diagonal” transfer function Gs(s), by considering W(s) as the decoupler transfer function. This document utilizes dynamic decoupling. The overall pre-compensator arrangement is shown in Fig 7.

**Dynamic decoupling** is a pre-compensator technique that uses the inverse of the system transfer function as a pre-compensator, decoupling the system over the entire frequency results in the following decoupler.


W(S)=[10−4(S^2+0.396*S−0.0359)0.018*S^3+1.95*S^2+45.49*S+2285.56(S^2+0.396*S−0.0359)0.129*S^2+75.35*S+2.588(S^2+0.396*S−0.0359)0.129*S^2+75.35*S+2.58810−4(S^2+0.396*S−0.0359)0.053*S^3+5.898*S^2+150.8*S+41.16]

Implies that;

$$W(S)=[\begin{array}{cc} D11 & D12\\ D21 & D22 \end{array}]$$ (21)

Decouplers for designing over the Simulink model of the overall system we used the following; since dynamic decoupling is available for decoupling the plant.

**Therefore;** the Decouplers are D_11,_ D_12,_ D_21_ and D_22_:

D11=10−4(S^2+0.396*S−0.03590.018*S^3+1.95*S^2+45.49*S+2285.56) (22)

D12=S^2+0.396*S−0.03590.129*S^2+75.35*S+2.588 (23)

D21=S^2+0.396*S−0.03590.129*S^2+75.35*S+2.588 (24)

D22=10−4(S^2+0.396*S−0.03590.053*S^3+5.898*S^2+150.8*S+41.16) (25)

**Fig 8:**
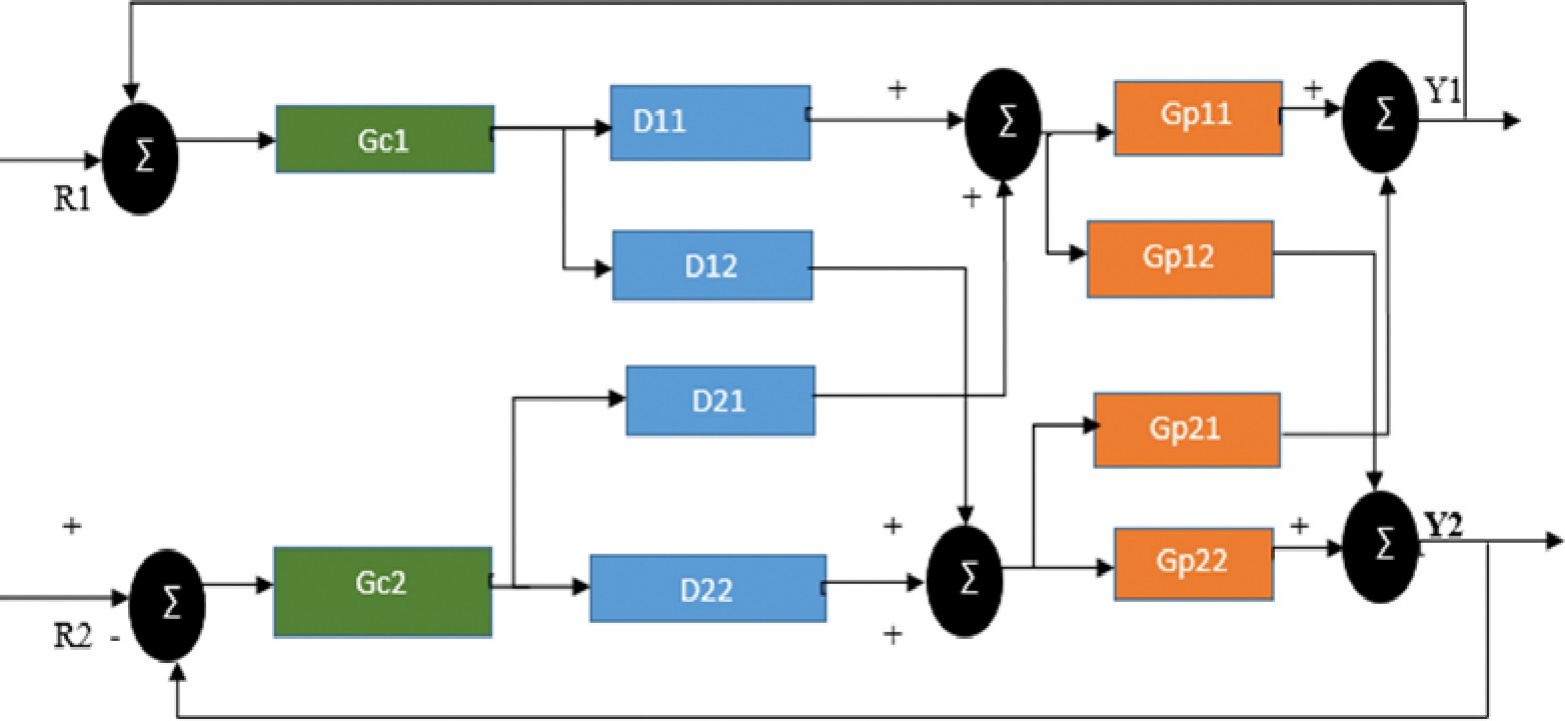
Decoupling control scheme with 1-1/2-2 pairing.

In the control scheme of the Fig 8 given above, there are two feedback controllers Gc_1_ and Gc_2,_ and four decoupling controllers D_11,_ D_12_, D_21,_ and D_22_. The Decouplers D_ij_, i, j = 1…2, have the role of cancelling the interaction effect from input j on the output i.

#### 3.2.2 Checking the possibility of controlling the system (using CN)

Let’s first investigate the possibility of controlling the system using CN (condition number) at steady state operation before designing controllers (using MATLAB command). The transfer function at zero frequency is:

$$G0=[\begin{array}{cc} \frac{5.3*10^{-4}}{-0.000196} & \frac{4.95}{-0.00066}\\ \frac{4.95}{-0.00066} & \frac{4.6*10^{-4}}{-0.000196} \end{array}]=[\begin{array}{cc} -27040.82 & -7500\\ -7500 & -23469.38 \end{array}]$$ (26)

Using MATLAB command, first, insert the transfer function at zero frequency and then determine the singular values using the singular value decomposition (SVD) command and evaluating the condition‐number (CN):

$$CN=\frac{Maximum\sin\;gularvalue}{minimum\sin\;gularvalue}=\frac{3.2965*10^{-4}}{1.7545*10^{-4}}=1.8789<10$$ (27)

This small condition number demonstrates that the system is in good condition implies possible to control the system.

### 3.3 Fuzzy Logic Controller (FLC) design and Genetic Algorithm (GA)

#### 3.3.1 Fuzzy membership functions design

FL applications have been gaining a high speed ever since the Japanese started using them in commercially available appliances. Nowadays, it is possible to find fuzzy-based applications in almost every area [12].

Fuzzy logic systems are widely used for control, system identification, pattern recognition problems, and many more applications from industry to academia. The membership functions (MFs) play vital role in the overall performance of fuzzy representation. The MFs are the building blocks of fuzzy set theory, that is, fuzziness in a fuzzy set is determined by its MF. Accordingly, the shapes of MFs are important for a particular problem since they effect on a fuzzy inference system. They may have different shapes such as triangular, trapezoidal, Gaussian, and so forth. The only condition a MF must really satisfy is that it must vary between 0 and 1.

The membership function is a graphical representation of the magnitude of participation of each input variable. The number 1 assigned to an element means that the element is in the set s, and 0 means that the element is definitely not in the set S. All other values mean a graduated membership of the set S.

Membership functions representing fuzzy sets have different shapes, which are defined by certain types of mathematical formulas. The most used membership function types are triangular, trapezoid, bell, sinusoid, Gaussian. Cauchy and sigmoid. When using different MFs for a given problem, it has usually been found that triangular and Gaussian MFs have very good results, better than other types of MFs. The MFs can be of any shape and form as long as it maps the given data with desirable degree of memberships. As far as choice of MFs is concerned, it is us to decide. This is where fuzzy system offers individual degrees of freedom. With experience, one will come to know which shape of MF is good for the application under consideration.

As there are infinite number of ways to characterize fuzziness, there are infinite number of ways to graphically depict the MFs that describe this fuzziness. The choice of which of the methods to use depends entirely on the problem size and problem type. Instead of choosing the shape of MF, setting the interval and number of MFs are also very important. For instance, to model a temperature coupled Tank control system by fuzzy logic, it is really important to know how many MFs are needed (e.g., Very Low, Low, Normal, High and Very High) MF and also choosing the intervals of MFs. These two factors also have a great impact on the outcome of a fuzzy logic system.

In addition, looking at the distribution of the data is a good idea. Although, trial and error method are often used for MF shape, because there is no exact method for choosing the MFs. The shape of MFs depends on how one believes in a given linguistic variable. It is more a question of intuition then criteria. The only condition a MF must really satisfy is that it must vary between 0 and 1. The function itself can be an arbitrary curve whose shape we can define as a function that suits us from the point of view of simplicity, convenience, speed, and efficiency. Therefore, the type of MF does not play a crucial role in shaping how the model performs. However, the number of MF has greater influence as it determines the computational time. Hence, the optimum model can be determined by varying the type of MFs for achieving best system performance.

The triangular function specifically as an integral transform kernel function from which more realistic signals can be derived, for example in kernel system estimation. Based on extensive review on many literatures, it can be concluded that the triangular MF is widely used because of its simplicity. Using various MF for given problems, usually triangular MFs are found to be closely performing well and better than other types of MF.

There are many references giving directions of how to choose MF [13]. The basic problem with modelling a situation, is to break the 0–1 modelling. This can be done by using triangular MF. However, if the situation is complex and deep, we might need a special type of MF. For instance, if the problem at hand is a quantum mechanics problem, then a special MF is needed. In order to make the best choice, one needs a lot of “experience” with the given situation. This experience will tune up and best fit, the subjective choice of the researcher with the given reality. There is no objective way to do so. Thus, a high-fidelity intuition based on sufficient experience will give an acceptable answer.

Generally speaking, triangular MF is one of the most encountered MF in practice. Of highly applied MFs, the triangular MFs are formed using straight lines. These straight-line membership functions have the advantage of simplicity to implement and fast for computation. Beside to all the above statements, it’s obvius that the selection criteria is depend on the researcher interest/ authors view of point to the model/system we conducted, due to this we are selected the triangular MF for our study.

**Fig 9:**
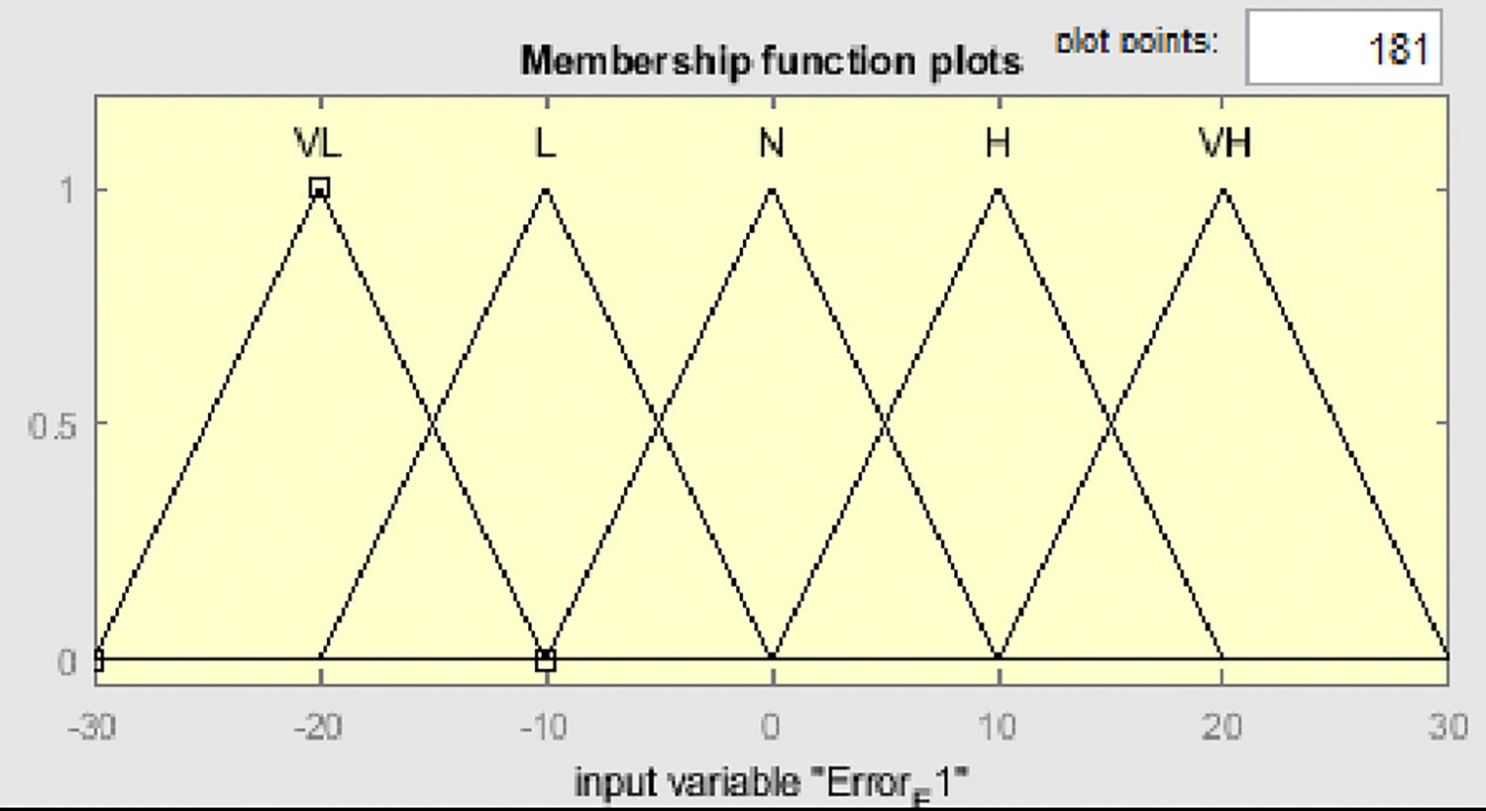
Membership function of error E1 for tank 1.

**Fig 10:**
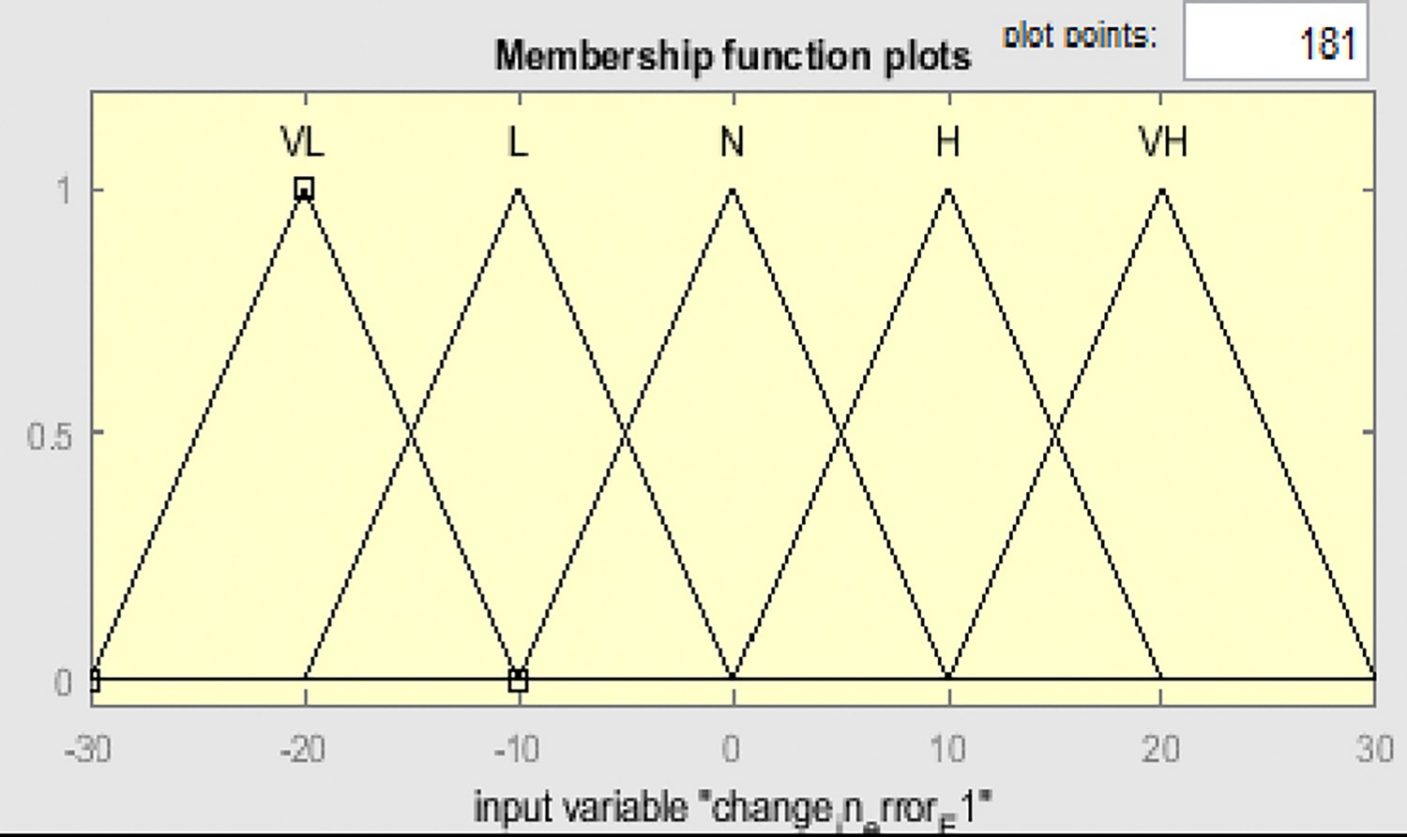
Membership function of change in error ΔE1 for tank 1.

**Fig 11:**
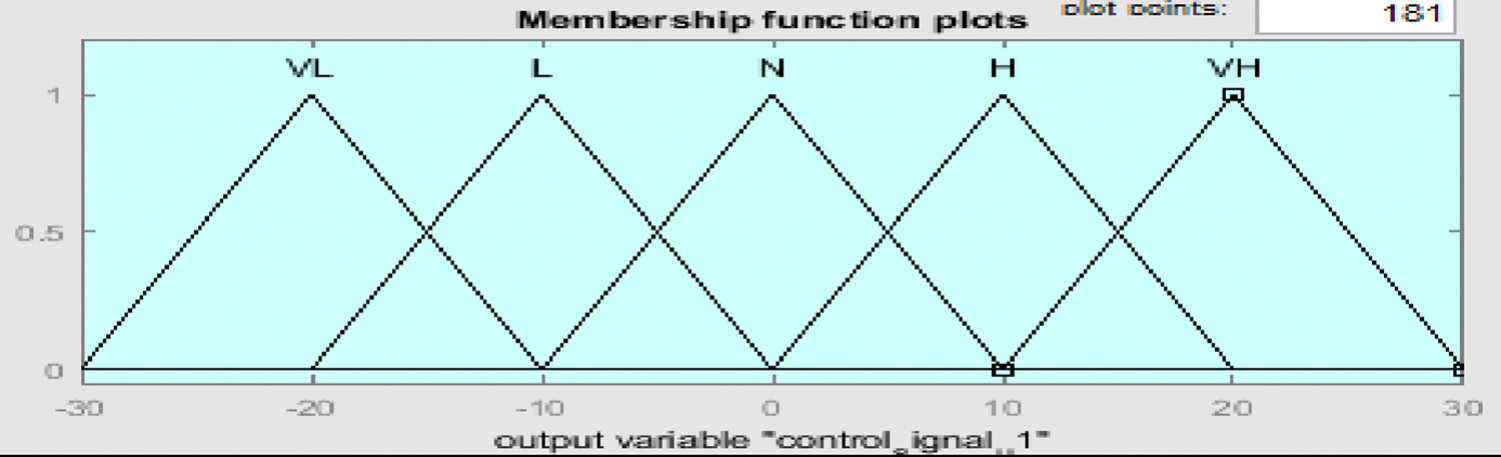
Membership function of output for tank 1.

**Fig 12:**
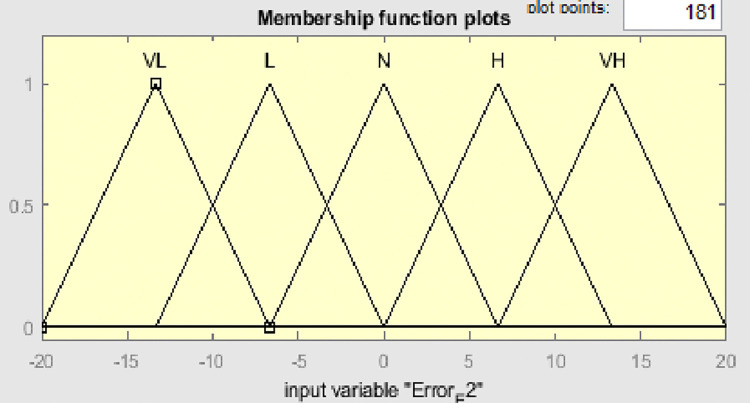
Membership function of error E2 for tank 2.

**Fig 13:**
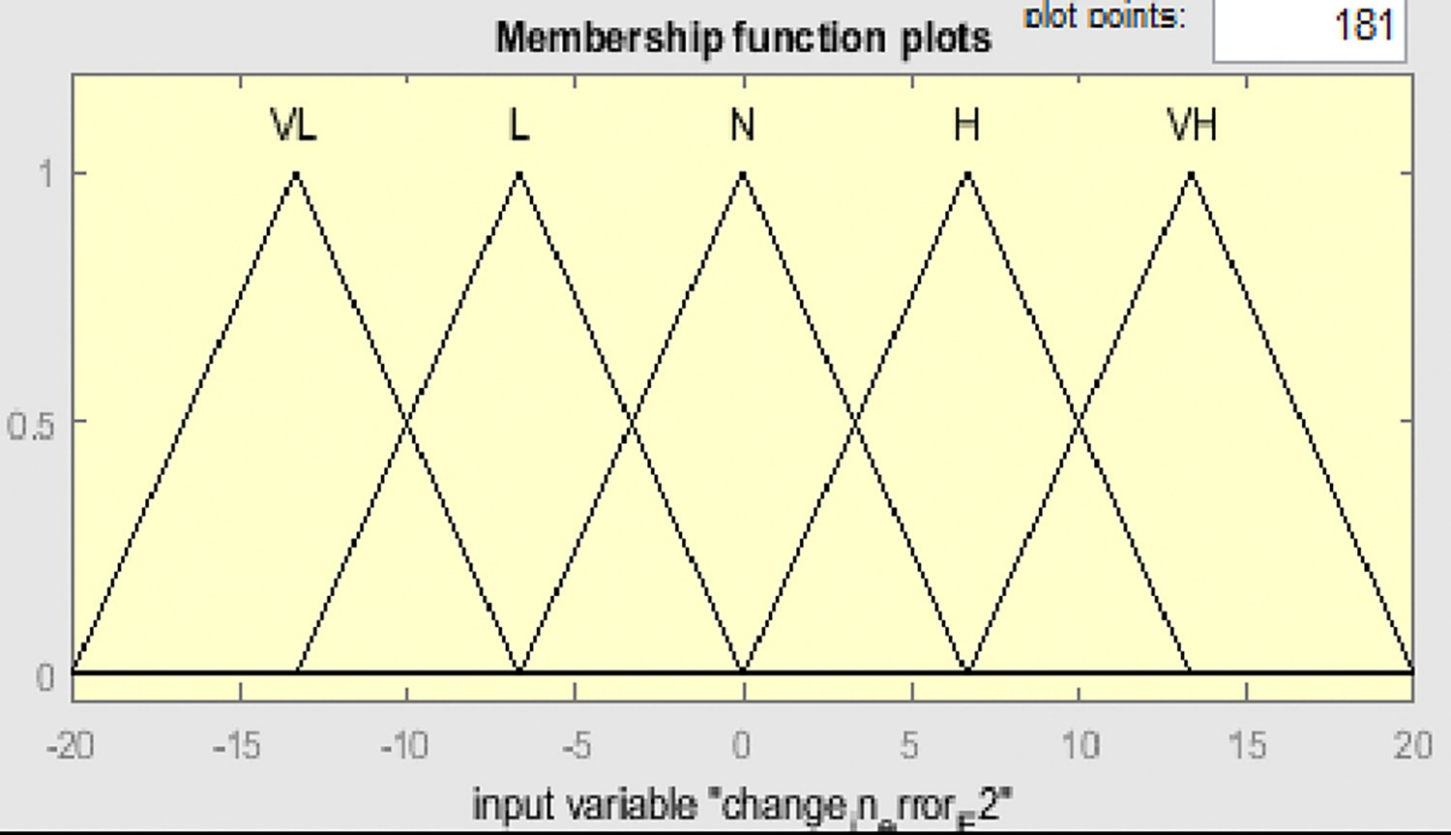
Membership function of change in error (ΔE2) for tank 2.

**Fig 14:**
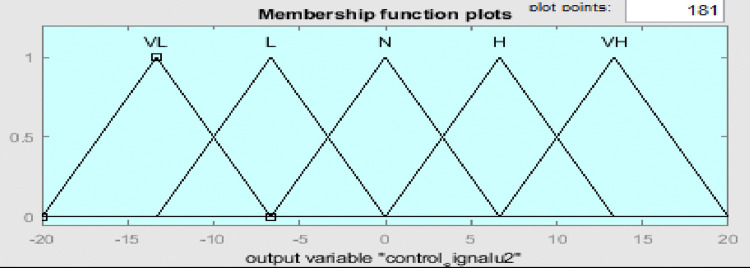
Membership function of output for tank 2.

Fuzzy membership functions range from 0 to 1, with five values: very low (VL), low (L), normal (N), high (H), and very high (VH). Figs 9–11 show error input, change in input, and output functions for tank 1, and Figs 12–14 show error input, change in input, and output functions for tank 2 respectively.

**3.3.2 Fuzzy reasoning/rule base and inference mechanism design.** The main idea of the rules is to bring a set point to its specified level point.

**Rule 1**: If E is H and ΔE is VH then output is VH. This implies that if the flow rate is very high then the valve is close fast.

**Rule 2**: If E is H and ΔE is H Then output is N. This implies that if flow is OK, and flow rate is OK then the valve need not be varied.

**Fig 15:**
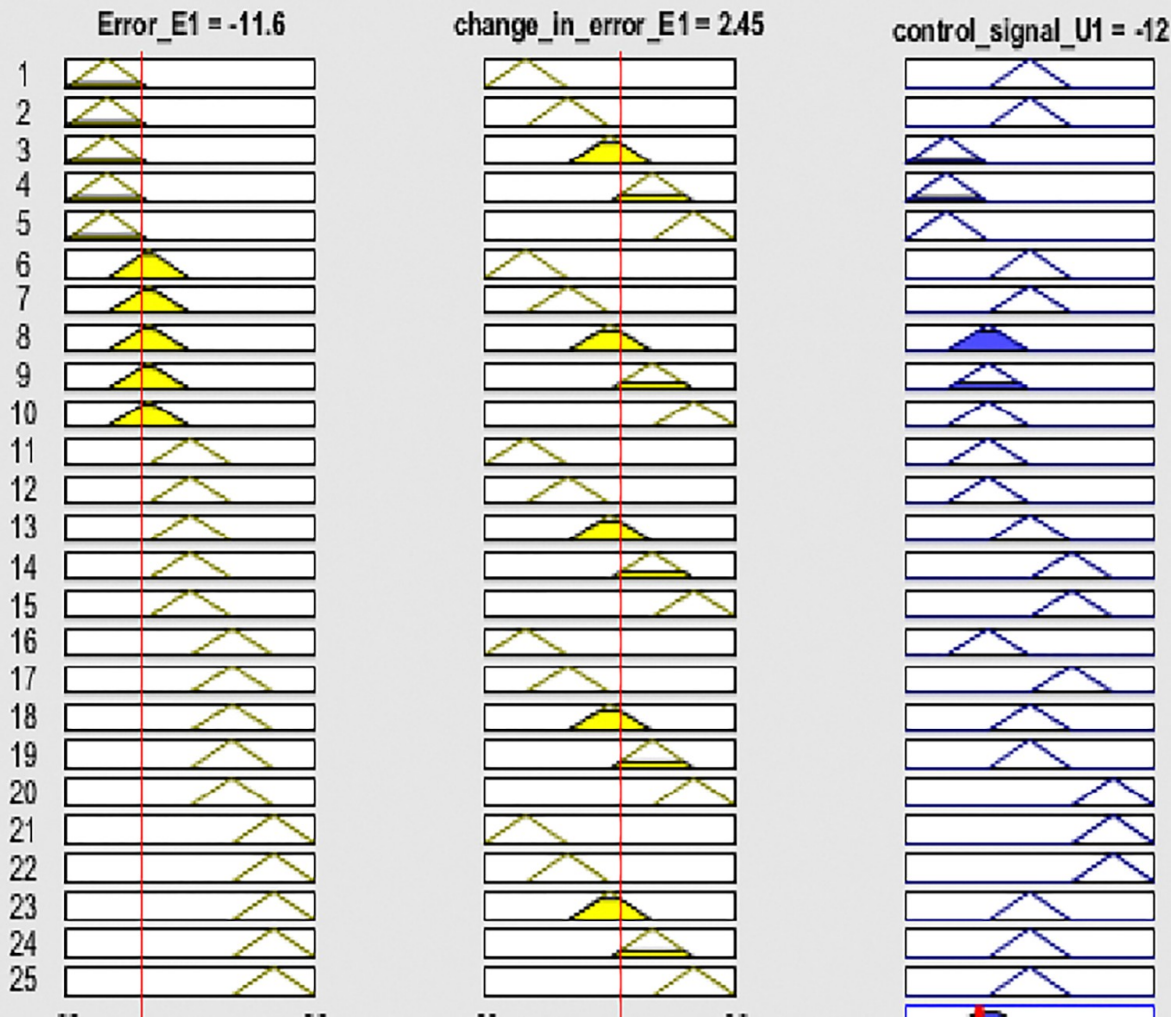
Rule viewed result from a set of rules for tank 1.

**Fig 16:**
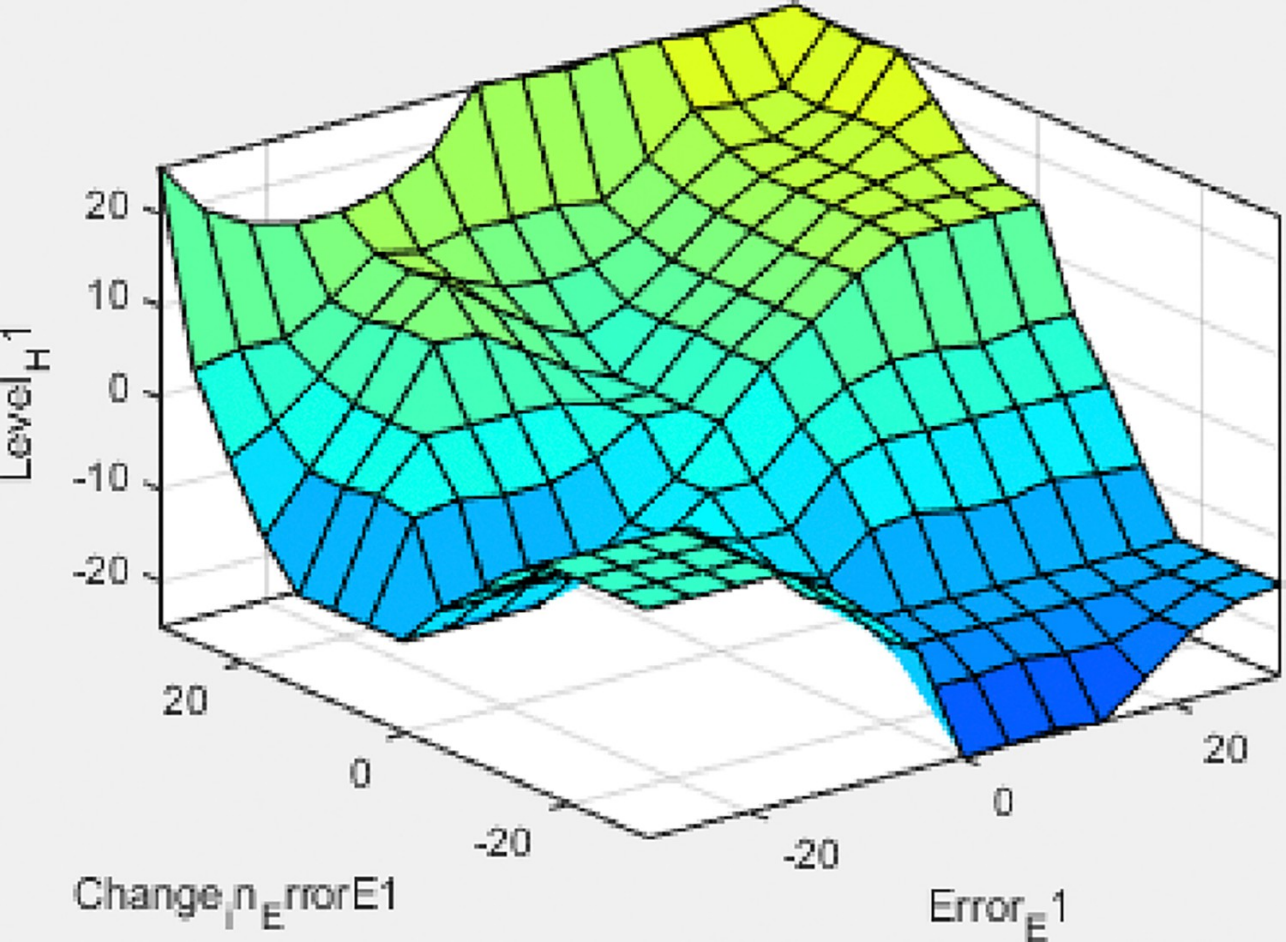
Surface viewed result from a set of rules for tank.

**Fig 17:**
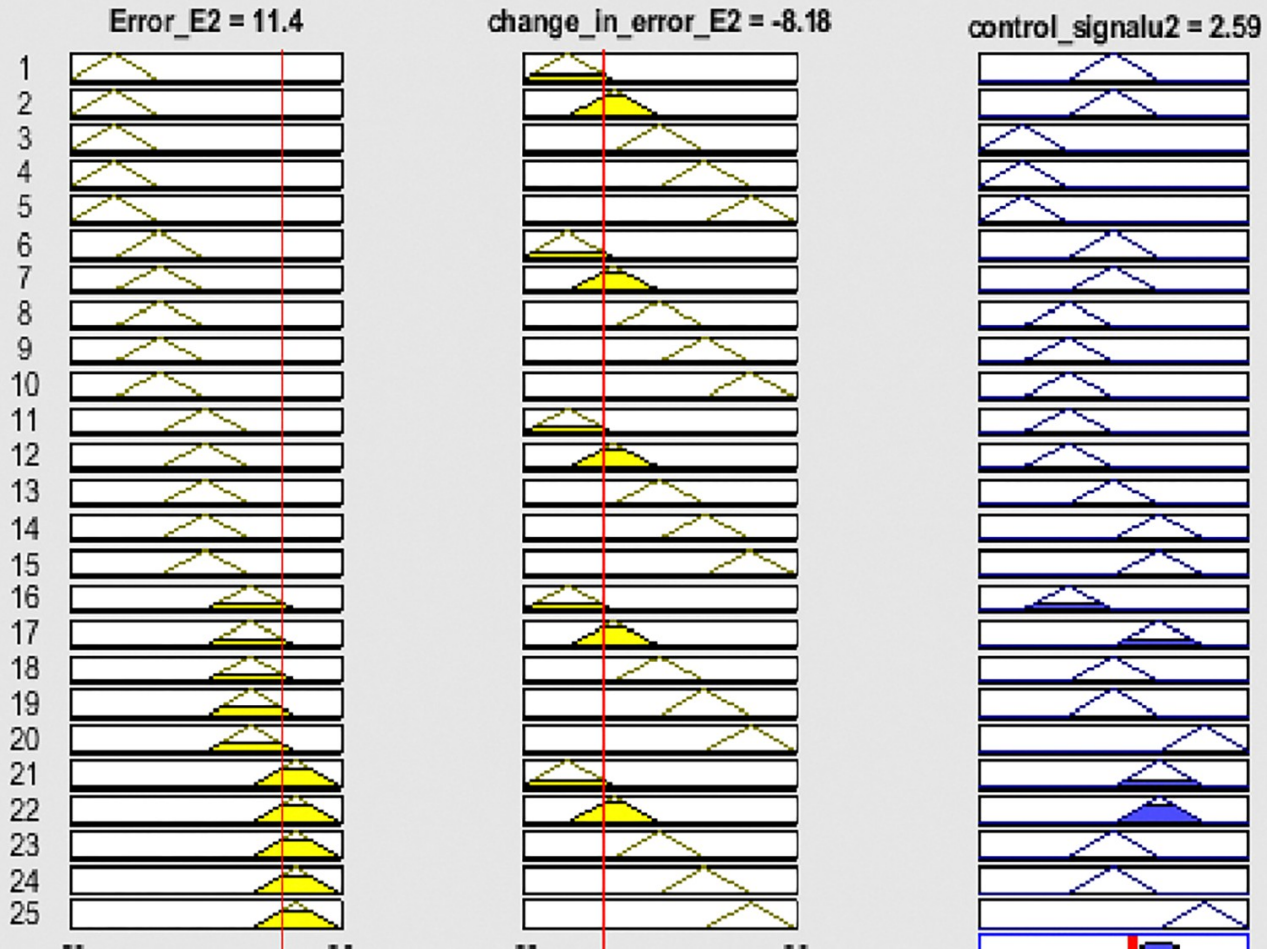
Rule viewed result from a set of rules for tank 2.

**Fig 18:**
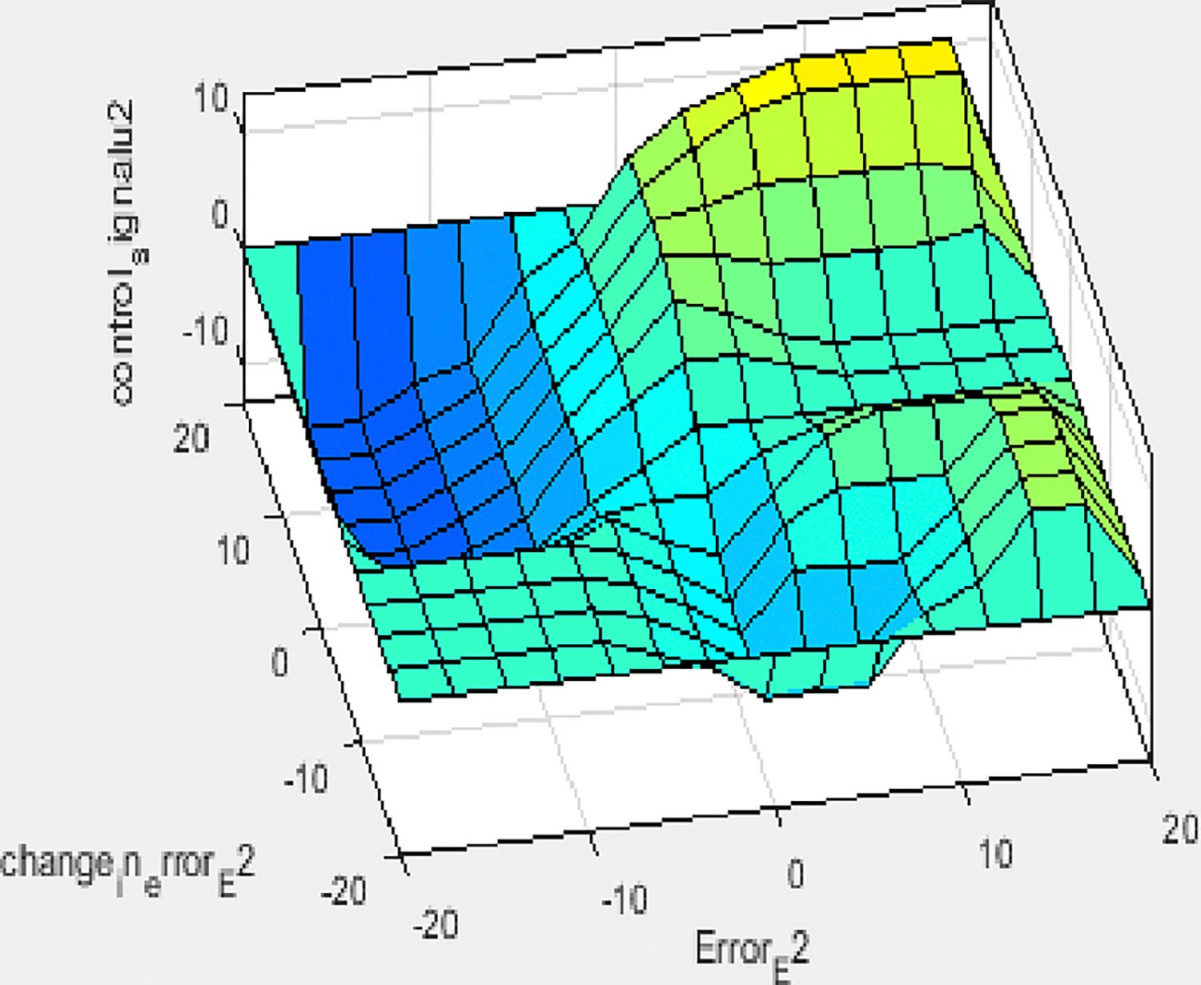
Surface viewed result from a set of rules for tank 2.

These rules are employed for controlling the liquid level such as it is reached to its specified value depending on membership functions mentioned above. The overall result of the rule viewed and surface viewed from the set of rules for tank 1 and the result of the rule viewed and surface viewed from the set of rules for tank 2 is shown below in Figs 15–18 respectively. As shown in the Table 3 below.

**Table 3:** Rules employed for controlling the system.

E ΔE	VL	L	N	H	VH
VL	N	N	VL	VL	L
L	N	N	L	L	L
N	L	L	N	N	H
H	L	H	N	N	VH
VH	H	VH	N	VH	VH

#### 3.3.3 Basic elements of GA (Genetic Algorithm)

The tuning of the GA-PID involves 6 variables, 20 population sizes, and 30 maximum generations which are all initially randomized for simulation/in-tuning algorithm. In determining the population of the new GA generation, where the fittest chromosome has one copy directly in the new generation. By interfacing the Simulink model with the codded algorithm of GA, the system employed for this paperwork is discussed in the next content.

### 3.4 MATLAB Simulink model and simulation results

Models for decoupling level and flow rate control of coupled tank systems are designed on the MATLAB/Simulink library. A plant model with FLC-PID and GA-PID controller is designed, and the result is verified.

**Fig 19:**
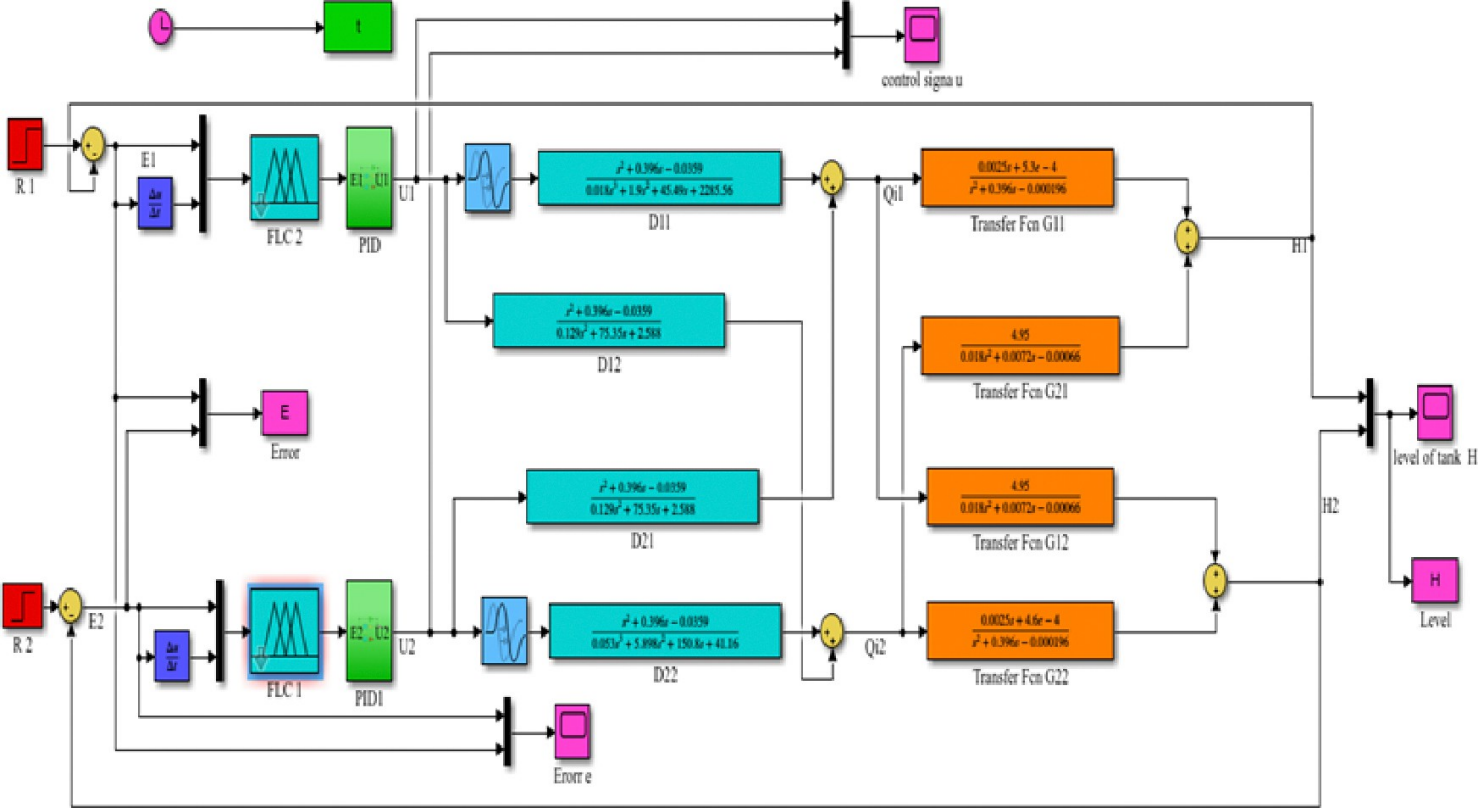
Simulink model of the decoupled system FLC with PID controller.

Fig 19 shown below is the MATLAB Simulink model and results for a decoupled system with an FLC- PID controller (Level response of the decoupled system for PID controller for tank1, H1, and tank2, H2).

#### 3.4.1 Simulink model and simulation result for the decoupled Simulink model for FLC with PID controller

**Fig 20:**
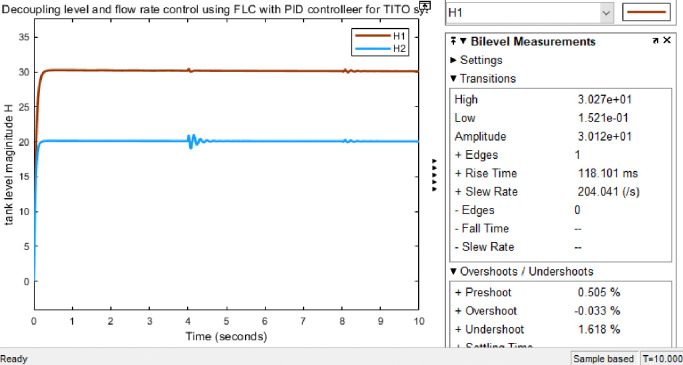
Simulation result for level of decoupled system for FLC with PID controller.

Figs 19 and 20 show that the Simulink model and the simulation result for the level of decoupled system for FLC with PID controller respectively; it has more results than the conventional PID controller. As the result tells us it has a fast rise time (118.101ms), short settling time (8.65 sec), and with no overshoot (-0.033%), undershoot (1.618%) for H1.

#### 3.4.2 Simulink model and simulation result for the decoupled Simulink model for FLC with PID controller with disturbance (robustness)

**Fig 21:**
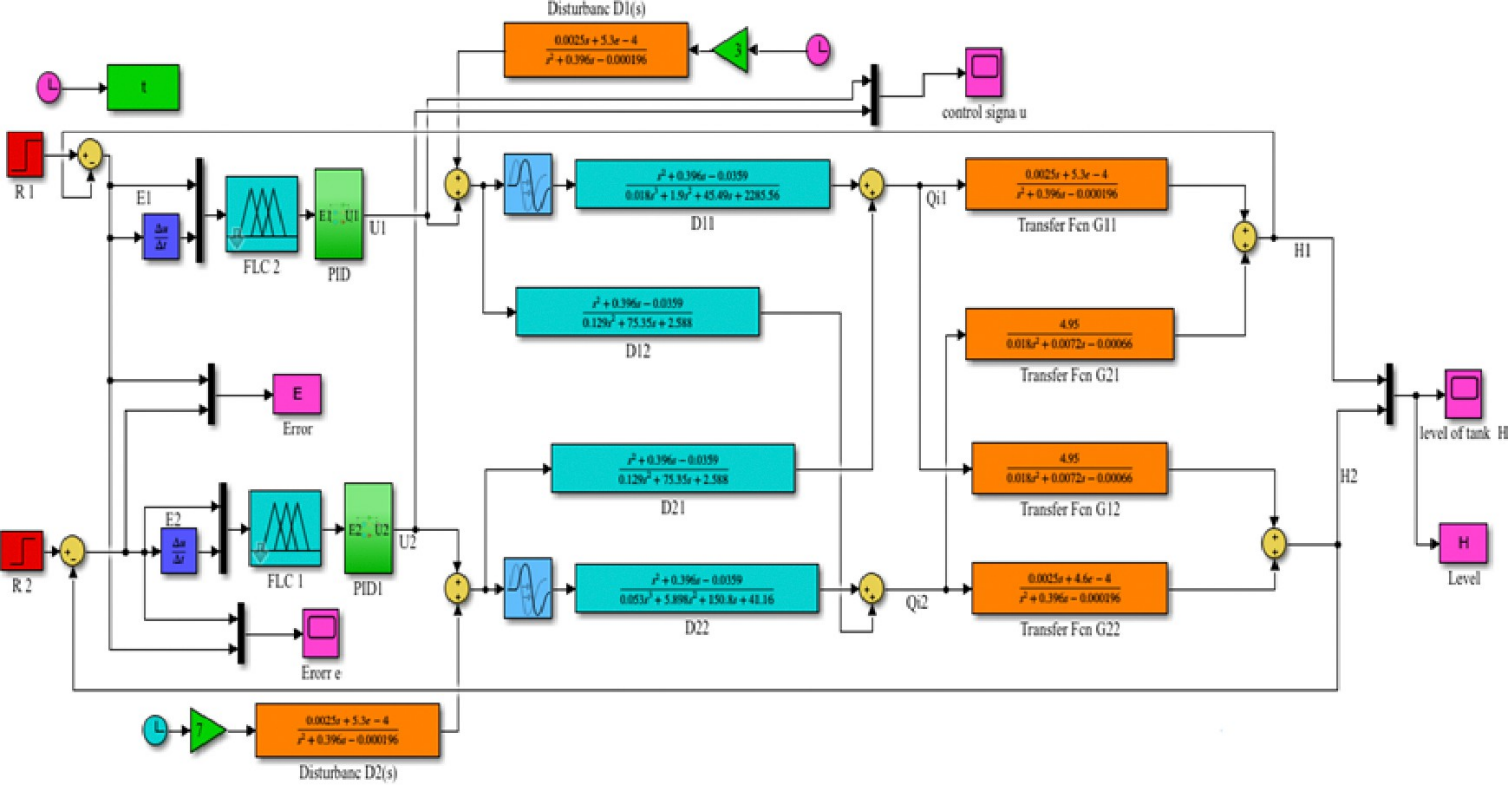
Simulink model of the decoupled system FLC—PID controller for robustness.

**Fig 22:**
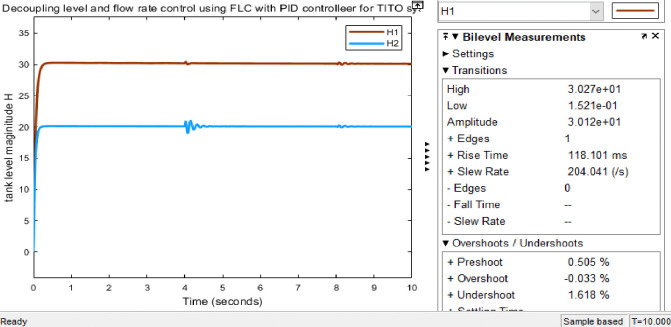
Simulation result for level of decoupled system for FLC with PID controller with disturbance.

As shown in Figs 21 and 22 Simulink model and simulation result of the FLC-PID controller tolerate disturbance (robust against disturbance) respectively. All the requirements of parameters for performance evaluation taken for FLC-PID without disturbance are the same with the presence of disturbance; like rise time (118.101ms), settling time (8.65sec), and with no overshoot (-0.033%)/undershoot (1.618%) for H1 as we see from the two response graphs.

#### 3.4.3 Simulink model and simulation result for the decoupled Simulink model for GA with PID controller

**Fig 23:**
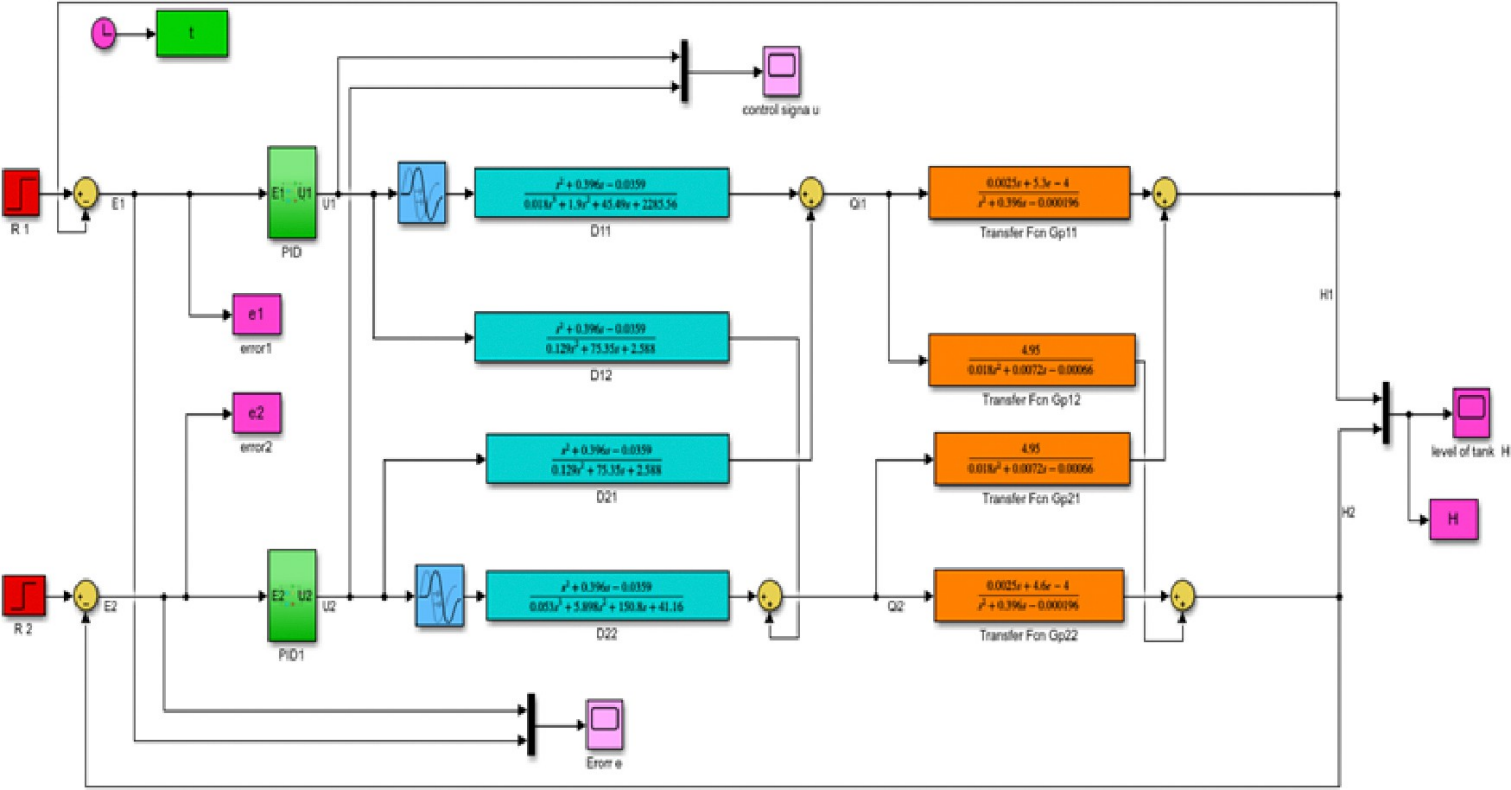
Simulink model of the decoupled system GA with PID controller.

**Fig 24:**
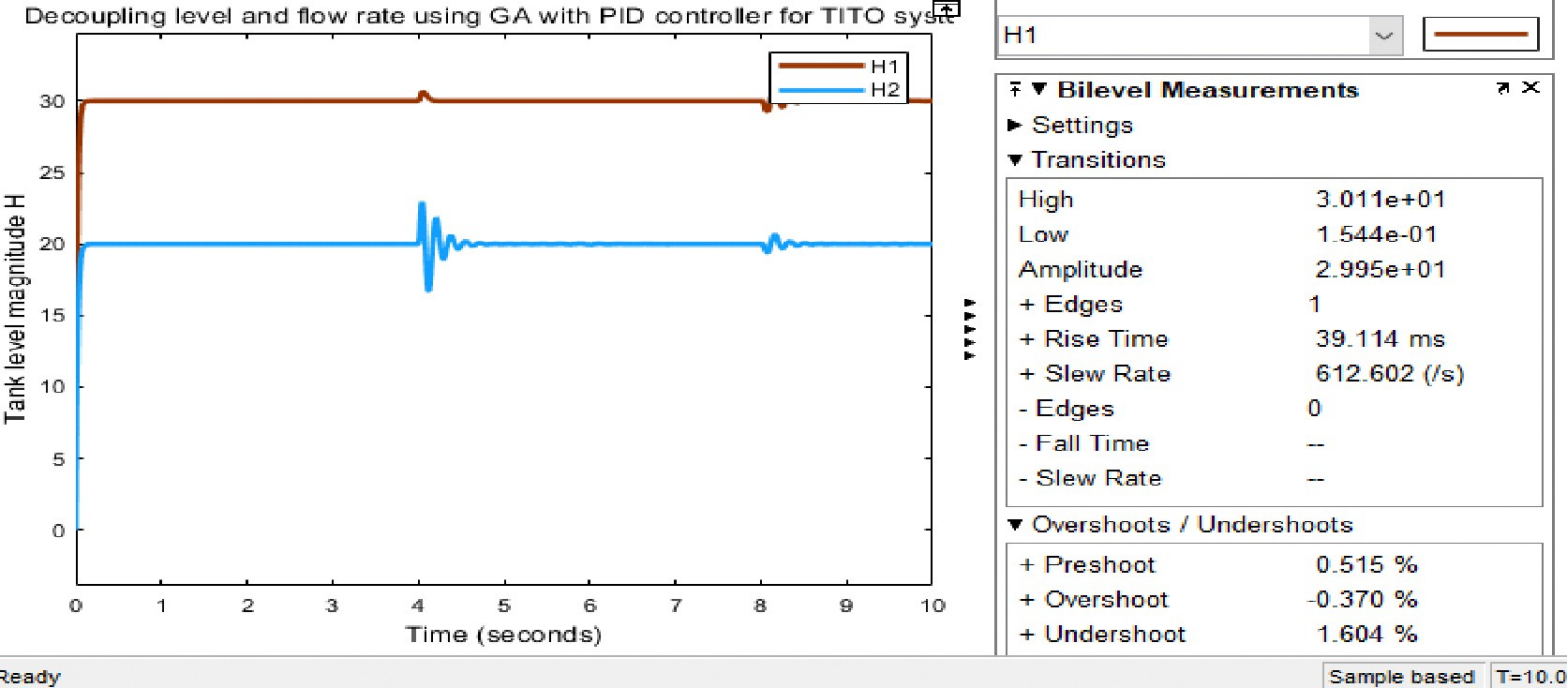
Simulation result of tank level H of the decoupled system GA with PID controller.

Figs 23 and 24 show the Simulink model and the response graphs of decoupled level and flow rate control using GA with PID controller for TITO system respectively. As the result shown in Fig 24 it has a fast rise time (39.114ms), short settling time (8.50sec), and overshoot (-0.370%)/undershoots (1.604%) for H1 is proven. It tells GA-PID has got more results than the rest.

#### 3.4.4 Simulink model and simulation result for the decoupled Simulink model for GA with PID controller with disturbance (robustness)

**Fig 25:**
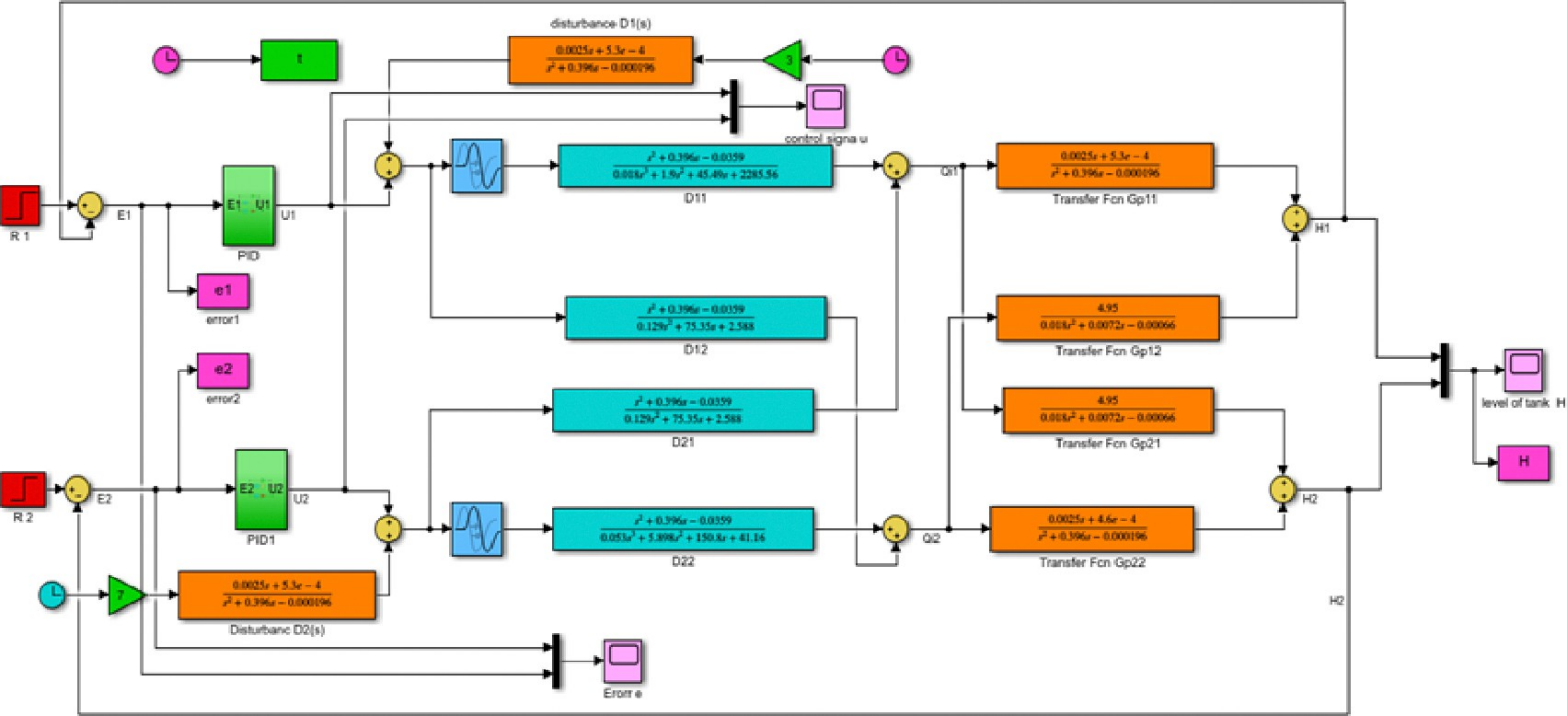
Simulink model of the decoupled system GA with PID controller for robustness.

**Fig 26:**
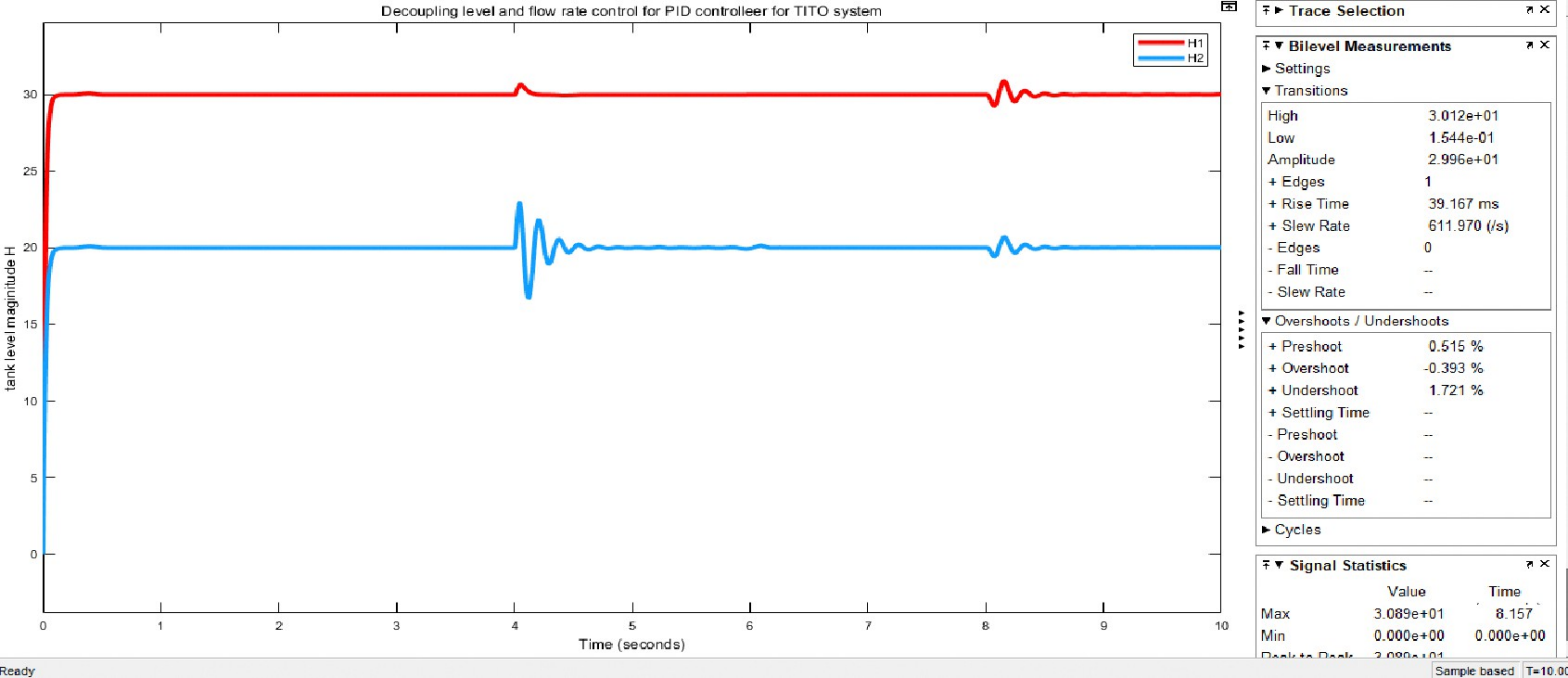
Simulation result of tank level H of the decoupled system GA -PID for robustness.

Figs 25 and 26 show; that the GA-PID controller tolerates disturbance, which are the input disturbances (robust against disturbance) to some extent Simulink model and results respectively. The requirements of parameter for performance evaluation taken for GA-PID without disturbance is the same with the presence of disturbance for settling time which is (8.50 sec). However, rise time (39.167ms) and overshoot (-0.393%)/undershoot (1.721%) with the presence of disturbance for H1, whereas rise time (39.114ms) and overshoot (-0.370%)/undershoot (1.604%) for H1 is proven. Here there are slight differences in measurements taken for evaluating the performance of the system.

### 3.5 Time domain performance indices for a set of controllers

Here in Table 4 shows below are time domain performance indices for a set of controllers that are taken for the sake of discussing the comparative study to ensure the objective of the paper stated above. Here the rise time, overshoot, and settling time are more important for the comparative study; while PID parameters are very vital for the control application.

**Table 4:** Time domain performance indices for a set of controllers.

Controllers	Tanks	Kp	Ki	Kd	Rise time (in ms)	OverShoot (in %)	Undershoot (in %)	Settling time (in ms)	Peak time (in sec)
Fuzzy-PID	Tank 1	9.59	0.951	0.552	118.101	-0.033	1.618	8.65sec	4.204
	Tank 2	12.56	0.850	0.536	101.497	-0.052	1.562	8.75sec	4.148
GA-PID	Tank 1	49.76	0.150	0.599	39.167	-0.393	1.721	8.50sec	0.038
	Tank 2	41.19	0.048	0.553	44.801	-0.338	1.955	8.52sec	4.017

### 3.6 Optimal control theory

Optimal control theory deals with the problem of finding a control law for a given system such that a certain optimality criterion is achieved. It is an extension of the calculus of variations and is a mathematical optimization method for deriving control policies. The method is largely due to the work of Lev Pontryagin and Richard Bellman in the 1950s, after contributions to the calculus of variations by Edward J. McShane. Optimal control can be seen as a control strategy in control theory [13].

According to optimal control, the objective of **minimum-time optimal operation** is to minimize the time required to drive a system from its initial state to the final state or to drive the system to the required desired set point. J or performance function is:

$$J=\int_{t0}^{tf}dt$$ (28)

For the **GA-PID** controller; the performance function J: For tank 1 and tank2 respectively:

$$J=\int_{t0}^{tf}dt=\int_{0}^{8.50}dt=\;8.50sec\;and\;J=\int_{t0}^{tf}dt=\int_{0}^{8.52}dt=\;8.52sec$$ (29)

For the **Fuzzy-PID** controller; the performance function J: For tank1 and tank2 respectively:

$$J=\int_{t0}^{tf}dt=\int_{0}^{8.65}dt=\;8.65sec\;and\;J=\int_{t0}^{tf}dt==\int_{t0}^{8.75}dt=\;8.75sec$$ (30)

From this, we understand that the system with the GA-PID controller has the more optimal result or the minimum time optimal problem is more achieved for the GA-PID controller than the other controllers.

## 4. Conclusion and recommendation for future works

### 4.1 conclusions

In this paper, a comparative study of two different controllers is done for controlling liquid levels and flow rates in the coupled tank system. The coupling system is a non-linear process. It can’t be controlled by a single controller. Hence two controllers are designed for each tank. To minimize the interactions, decouplers and controllers are also designed. Here, a comparative study of different Conventional with Intelligent Controller; FLC with PID, and GA with PID controllers are studied. Numerical simulation indicates that the FLC with PID controller has more advantages than the GA with PID controller.

Performance analyses of FLC with PID and GA with PID controllers have been done. The result/performance in the two radical controllers, which is based on the transient and steady-state specifications, shows that the GA with PID controllers reduces undershoot but greater overshoot, lowers the rise time, settling time, and peak time and provides minimum time optimal operation when compared to FLC with PID controllers. However, GA with PID has a greater overshoot than FLC with PID; small overshoot with a small settling time and rise time; in GA with PID controllers, the external disturbance tolerance capability of the proposed scheme is proved for given step inputs, meaning robustness against external disturbance has slight difference and FLC-PID has perfectly achieved the robustness. So, it is observed that FLC with a PID-based model gives a more performance to GA with PID controllers of the system as mentioned in the simulation result above because robustness is more important than time performance.

### 4.2 Recommendation for future works

In the future, recommended that the nonlinear model be realized within the real plant Distributed Control System (DCS) to act as an observer of the process. Real-time systems can be implemented to get more accurate results by first evaluating experimental tests. From a practical perspective, the proposed strategy can be integrated with the existing controller (FLC with PID), without any modifications. For this matter, recommend using a solenoid valve at the coupling point.

Hence, Process control of large industrial plants has evolved through many stages. Initially, control would be from panels local to the process plant. However this required a large amount of human oversight to attend to these dispersed panels, and there was no overall view of the process. The next logical development was the transmission of all plant measurements to a permanently-staffed central control room. Effectively this was the centralisation of all the localised panels, with the advantages of lower manning levels and easier overview of the process. Often the controllers were behind the control room panels, and all automatic and manual control outputs were transmitted back to plant.

However, whilst providing a central control focus, this arrangement was inflexible as each control loop had its own controller hardware, and continual operator movement within the control room was required to view different parts of the process. With the coming of electronic processors and graphic displays it became possible to replace these discrete controllers with computer-based algorithms, hosted on a network of input/output racks with their own control processors. These could be distributed around plant, and communicate with the graphic display in the control room or rooms.

The introduction of DCSs to this existing system allows easy interconnection and re-configuration of plant controls and easy interfacing with other production computer systems. It enabled sophisticated alarm handling, introduced automatic event logging, removed the need for physical records such as chart recorders, allowed the control racks to be networked and thereby located locally to plant to reduce cabling runs, and provided high level overviews of plant status and production levels.

A **distributed control system** (**DCS**) is a computerized control system for a process or plant usually with many control loops, in which autonomous controllers are distributed throughout the system, but there is no central operator supervisory control. The DCS concept increases reliability and reduces installation costs by localizing control functions near the process plant, with remote monitoring and supervision.

## Supporting information

S1 File (DOCX)

## List of abbreviations

ANN: Artificial Neural Network
CN: Condition Number
COG: Center of Gravity
COAs: Center of Areas
CTS: Coupled Tank System
FLC: Fuzzy Logic Controller
FRF: Frequency Response Function
FOPDT: First Order Pulse Dead Time
GA: Genetic Algorithm
IAE: Integral of the Absolute Error
IMC: Internal Mode Control
MIMO: Multi Input Multi Output
MPC: Model Predictive Control
MSMPR: Mixed Suspension Mixed Product Removal
MPCANN: Model Predictive Control Artificial Neural Network
MRAC: Model Reference Adaptive Control
NGPC: Nonlinear Generalized Predictive Control
PID: Proportional Integral Derivative
SFLD: State Feedback Linearization with Decoupling
SISO: Single Input Single Output
TITO: Two Input Two Output
