# Supplementary material for: Decoupled level and flow rate control of a two-tank system in beverage production: A comparative analysis of Fuzzy-PID and GA-PID for minimum time operation
Source: PLoS One. 2025 Feb 5;20(2):e0317600. doi: 10.1371/journal.pone.0317600 (PMC11798464; doi:10.1371/journal.pone.0317600)
Supplement: S1 File — (DOCX) [file pone.0317600.s001.docx]

To:

The Editor-in-Chief,

Plos One

Dear Editor-in-Chief,

First of all, we would like to thank you for considering our paper entitled, “Decoupled Level and Flow Rate Control of a Two-Tank System in Beverage Production: A Comparative Analysis of Fuzzy-PID and GA-PID for Minimum Time Operation” by Tamiru Demelash Kassie, Mebratu Sintie Geremew, and Kassahun Ashagrie Chanie for the publication in your esteemed journal. We are here to upload the final revised manuscript of the paper prepared by taking all the comments and suggestions of the respected Referees into consideration. We strongly hope and believe that you will find the revised manuscript suitable for publication in your esteemed journal of Plos One. The point-wise Authors’ responses to the Referee's comments/suggestions are given in the following for your consideration and needful action in this regard.

Looking forward to receiving the earliest communication in this regard.

Thank you, with warm regards,

Mebratu Sintie Geremew and Team

Department of Electrical and Computer Engineering,

Mizan-Tepi University, Tepi, Ethiopia

Author's Response to Reviewers’ Comments

Article ID: PONE-D-24-21554

***Title: Decoupled Level and Flow Rate Control of a Two-Tank System in Beverage Production: A Comparative Analysis of Fuzzy-PID and GA-PID for Minimum Time Operation.***

Authors: Tamiru Demelash Kassie, Mebratu Sintie Geremew, and Kassahun Ashagrie Chanie. First of all, the authors would like to convey their sincere thanks and gratitude to the respected reviewers for their valuable comments and suggestions for the improvement of the technical quality of the manuscript. The authors have tried their level best to incorporate all the comments of the respected reviewers. We strongly hope that the quality of the revised manuscript has been improved enormously after taking the respected reviewers’ comments and suggestions into consideration.

**Minimal data set as a Supporting Information files:**

**Thank you for your suggestion. we have put minimal data sets as follow (set of parameter vales for obtaining mathematical modeling of the system in terms of manipulating variables and process variables), so that a set of state space equations with set of state space representation/matrix to further analysis (to get transfer function using MALAB commands, to decoupled the system, to build Simulink model, …).**

**The other data set is the MATLAB script (program for tuning and program simulation) as stated below:**

- **Set parameter values for mathematical modeling:**

Each value of β_1_, β_2_, β_3_, A_1_, A_2_, H_1,_ and H_2_ can be obtained from mathematical modeling equations as shown in Table 1.

Table 1: Parameter values [8]

| H_1_, H_2_ | 30 m, and 20 m respectively |
| --- | --- |
| $\beta1$ | 50 |
| $\beta2$ | 50 |
| $\beta3$ | 50 |
| $A1$ | 400 m^2^ |
| $A2$ | 400 m^2^ |

By using the Parameters value and equations (13) and (14), we can get the following equations in the form of manipulating variables q_1_, q_2_ and process variables h_1_, h_2_: -

## APPENDIX: MATLAB script

### B1: Program for tuning

function tune_tamega

clear all

options= optimoptions('ga','PlotFcn',@gaplotbestf);

options.PopulationSize= 20;

options.MaxGenerations= 30; % Increase value if needed

nvar= 6; % Number of parameters

LB= 0.0001*ones(1,nvar); % % Lower bound

UB= [50 2 2 50 2 2]; % Upper bound of tuning parameters

[x,fval]= ga(@EvalObj,nvar,[],[],[],[],LB,UB,[],options)

function obj= EvalObj(x)

% Updates the values of Kp1, Ki1, Kd1, Kp, Ki and Kd in model workspace

simulink_model= 'tamega';

load_system(simulink_model);

gains= get_param(simulink_model,'modelworkspace');

gains.assignin('Kp1', x(1));

gains.assignin('Ki1', x(2));

gains.assignin('Kd1', x(3));

gains.assignin('Kp', x(4));

gains.assignin('Ki', x(5));

gains.assignin('Kd', x(6));

% Simulates the simulink model to get the outputs

simOut= sim(simulink_model,'SaveOutput','on'); %Retrieves time t error e1 and error e2

t= simOut.find('t');

e1= simOut.find('e1');

e2= simOut.find('e2'); % Calculate a performance index

n= length(e1);

ae= abs(e1)+abs(e2); % IAE= int(|e1|+|e2|)

obj= 0;

for i= 2:n

stepsize= t(i)-t(i-1);

obj= obj+0.5*stepsize*(ae(i-1)+ae(i));

end

### B2: Program for simulation

%% Simulate the tuned PID control system for a step change

% The simulink model needs seven gains x= [Kp1 Ki1 Kd1 Kp Ki Kd].

x = [x1	x2	x3 ...

x4	x5	x6]; %fval = y

simulink_model= 'tamega';

load_system(simulink_model);

gains= get_param(simulink_model,'modelworkspace');

gains.assignin('Kp1', x(1));

gains.assignin('Ki1', x(2));

gains.assignin('Kd1', x(3));

gains.assignin('Kp', x(4));

gains.assignin('Ki', x(5));

gains.assignin('Kd', x(6)); % Simulates the simulink model to get the outputs

simOut= sim(simulink_model,'SaveOutput','on'); % Retrieves t and y

H= simOut.find('H');

t= H.Time;

y1= H.Data(:,1);

y2= H.Data(:,2);

subplot 211

plot(t,y1,'linewidth',2)

axis([0 t(end) 0 40])

xlabel('t'), ylabel('y1(t)')

subplot 212

plot(t,y2,'linewidth',2)

axis([0 t(end) 0 30])

xlabel('t'), ylabel('y2(t)')
